# Spreading depolarization suppression from inter-astrocytic gap junction blockade assessed with multimodal imaging and a novel wavefront detection scheme

**DOI:** 10.1016/j.neurot.2023.10.008

**Published:** 2023-12-19

**Authors:** Dene Ringuette, Azin EbrahimAmini, Weerawong Sangphosuk, Mark S. Aquilino, Gwennyth Carroll, Max Ashley, Paolo Bazzigaluppi, Suzie Dufour, Marine Droguerre, Bojana Stefanovic, Ofer Levi, Mathieu Charveriat, Philippe P. Monnier, Peter L. Carlen

**Affiliations:** a Department of Physiology, Faculty of Medicine, University of Toronto, 1 King's College Circle, Toronto, Ontario M5S 1A8, Canada; b Division of Genetics and Development, Krembil Research Institute, 60 Leonard Ave., Toronto, Ontario M5T 2S8, Canada; c Krembil Neuroscience, Krembil Research Institute, 60 Leonard Ave., Toronto, Ontario M5T 2S8, Canada; d The Institute Biomedical Engineering, University of Toronto, 164 College St., Toronto, Ontario M5S 3G9, Canada; e The Edward S. Rogers Sr. Department of Electrical and Computer Engineering, University of Toronto, 10 King's College Rd., Toronto, Ontario M5S 3G4, Canada; f Department of Ophthalmology & Vision Science, Faculty of Medicine, University of Toronto, 340 College St., Toronto, Ontario M5T 3A9, Canada; g Department of Medical Biophysics, University of Toronto, 610 University Ave., Toronto, Ontario M5G 2M9, Canada; h Sunnybrook Health Sciences Center, 2075 Bayview Ave., Toronto, Ontario M4N 3M5, Canada; i Theranexus, 60 Ave. Rockefeller, 69008, Lyon, France

**Keywords:** Spreading depolarization, Multimodal imaging, Potassium recording, Pharmacological intervention, Gap junction blockers

## Abstract

Spreading depolarizations (SDs) are an enigmatic and ubiquitous co-morbidity of neural dysfunction. SDs are propagating waves of local field depolarization and increased extracellular potassium. They increase the metabolic demand on brain tissue, resulting in changes in tissue blood flow, and are associated with adverse neurological consequences including stroke, epilepsy, neurotrauma, and migraine. Their occurrence is associated with poor patient prognosis through mechanisms which are only partially understood. Here we show *in vivo* that two (structurally dissimilar) drugs, which suppress astroglial gap junctional communication, can acutely suppress SDs. We found that mefloquine hydrochloride (MQH), administered IP, slowed the propagation of the SD potassium waveform and intermittently led to its suppression. The hemodynamic response was similarly delayed and intermittently suppressed. Furthermore, in instances where SD led to transient tissue swelling, MQH reduced observable tissue displacement. Administration of meclofenamic acid (MFA) IP was found to reduce blood flow, both proximal and distal, to the site of SD induction, preceding a large reduction in the amplitude of the SD-associated potassium wave. We introduce a novel image processing scheme for SD wavefront localization under low-contrast imaging conditions permitting full-field wavefront velocity mapping and wavefront parametrization. We found that MQH administration delayed SD wavefront's optical correlates. These two clinically used drugs, both gap junctional blockers found to distinctly suppress SDs, may be of therapeutic benefit in the various brain disorders associated with recurrent SDs.

## Graphical abstract

**Figure:**
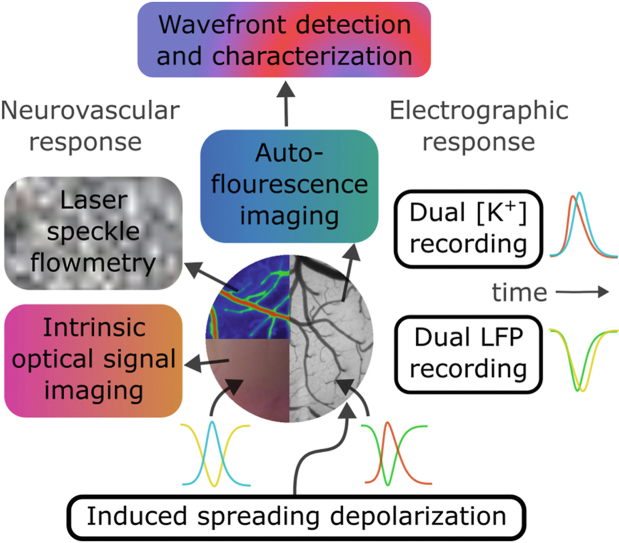

## Introduction

Spreading depolarizations (SDs) are propagating waves of depolarization within brain tissue accompanied by increased extracellular potassium ([K^+^]_e_) and various metabolic changes. Sufficiently large SDs silence neural activity, and are then called spreading depression [1]. SDs and spreading depression are associated with poor clinical prognosis in several disease states, including stroke [2], seizures [3], and traumatic brain injury (TBI) [4]. Furthermore, SD is associated with sudden unexpected death in epilepsy (SUDEP) [5,6]. In the context of cerebral ischemia, recurrent SDs are seen as a continuum from a non-damaging response to the cause of spreading necrosis [7,8]. Consequently, we are motivated to characterize SD in the mouse cerebral cortex and examine the effects of two clinically used drugs, meclofenamic acid (MFA) and mefloquine hydrochloride (MQH), for potential clinical utility. Moreover, we are motivated to concurrently investigate factors related to brain ischemia/hypoxia such as cerebral blood flow and tissue oxygenation.

Anti-migraine drugs reduce SD susceptibility [9]. The chronic administration of lamotrigine suppresses SD propagation and induction frequency [9]. Conversely, chronic administration of valproate has no effect on induction but reduces event propagation likelihood and velocity [9]. Additionally, ketamine has been shown in cases to transiently block or reduce occurrence [10,11] but is limited by tolerance [12,13]. We chose to investigate the acute effects of drugs which can be administered chronically in the clinical setting. MFA is a non-steroidal anti-inflammatory agent [14] and an anticonvulsant, which opens KCNQ2/Q3 potassium channels [15] and blocks interastrocytic Cx30 and Cx43 gap junctional communication [16,17]. The KCNQ2 gene belongs to the family of K_V_7.2 channels. The anti-malarial drug MQH blocks K_V_7.1 channels [18] and also Cx36 (neurons) and Cx50 (horizontal cells) gap junctional channels [19]. More recently, MQH has been found to block Cx43 (astrocytic) gap junctions synergistically with amitriptyline [20]. In this work, we show that these two drugs, both capable of blocking interastrocytic gap junctional communication, can acutely suppress SD by different mechanisms.

Evidence has shown that neurons and not astrocytes are the primary drivers of the major components of SD [21]. This includes intracellular acidification, mitochondrial depolarization, fast-phase intracellular calcium spiking, and transient swelling. However, given that potassium is the critical driver of SD propagation and that potassium buffering is mediated by astrocytes [[22], [23], [24], [25]], an optimal target for depolarization suppression may be astrocytic gap junctional communication. We have recently characterized several modulators of astrocytic gap junctions on the propagation of SD in mouse cortex *in vivo* [1]. These results show that astrocytic gap junctions alter the kinetics of [K^+^]_e_ propagation leading to changes in depolarization waveforms. More direct modulation of potassium and metabolic dynamics in astrocytes have been shown to effect SD. Particularly, ciliary neurotrophic factor activation of astrocytes has been shown to decrease SD susceptibility and increase potassium clearance [26]. Moreover, astrocytic metabolism has been shown to exert regulation over SD events [27]. Furthermore, astrocytes have a multifaceted role in SD and have been investigated as a target for ​limiting SD [26].

In this work, we used an *in vivo* multi-channel [K^+^]_e_ and local field potential (LFP) recording scheme with concurrent multi-modal imaging of blood flow, blood oxygenation, and autofluorescence (AF) to investigate SD dynamics under conditions of MFA and MQH modulation. Combined with novel image filtering, we were able to investigate the broader dynamics of SD propagation. We suggest the novel concept that a major contributor to the clinical affect of MFA and MQH is through the modulation of glial communication. This is supported by our finding that these drugs modify neuro-glial ion diffusion [28]. Here, we show that these two drugs administered to block astroglial gap junctional communication can acutely suppress SD propagation. Their respective effects on wave propagation dynamics are characterized using a novel image processing scheme. We discovered a high degree of inter-event variability in SD spatiotemporal dynamics, motivating the need for concurrent imaging when assessing SD-associated ion movement.

## Methods

### Electrophysiology and imaging system

**Fig. 1:**
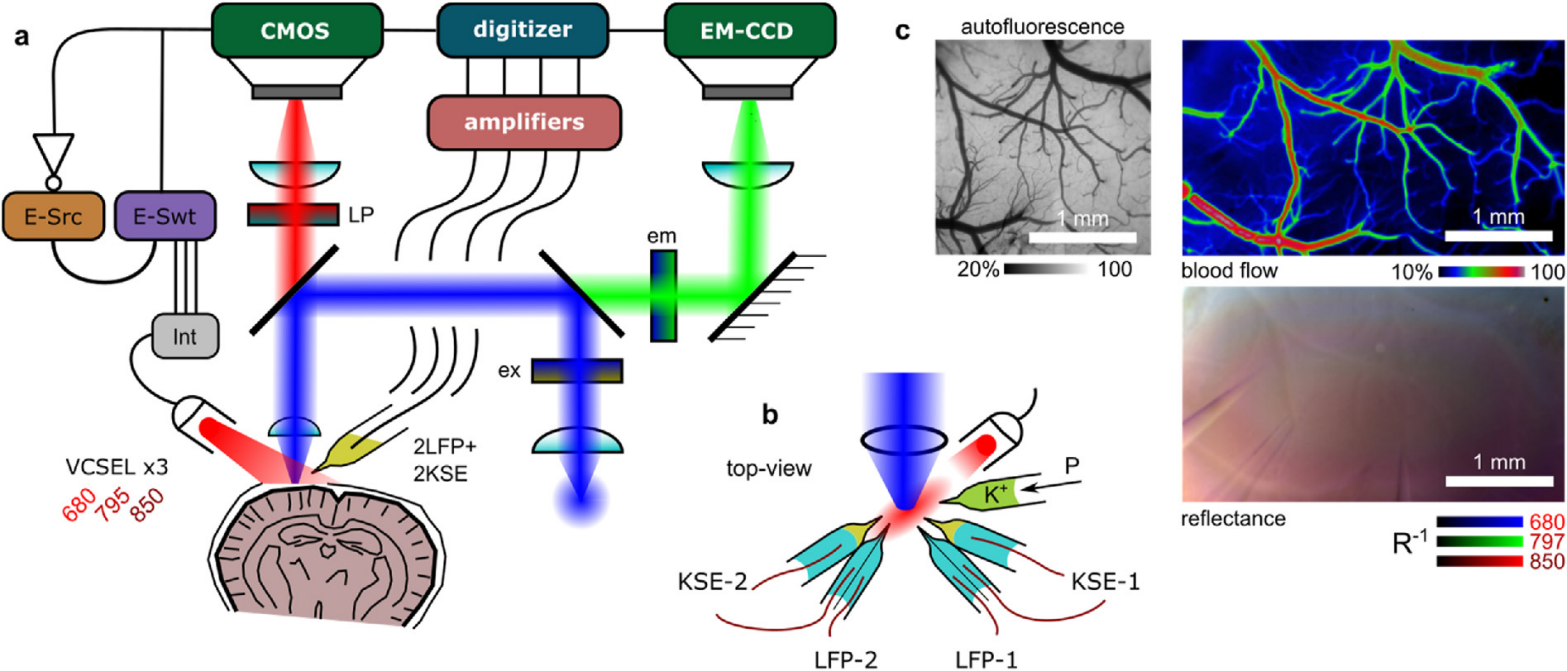
Multi-modality rodent optical imaging system. **a)** System diagram showing separate light paths and synchronization with LFP recording. *Legend:* CMOS and EM-CCD: cameras for NIR imaging and autofluorescence (AF), respectively, E-Src & E-Swt: current source and switch, respectively, Int: interface adaptor, Ex/Em: AF band-pass filters, LP: long-pass filter and dichroic mirror, Int: pin adapter interface, and VCSEL for NIR illumination module. **b)** Electrode and illumination source arrangement on cortex. **c)** The FOV for LSCI, IOSI and AF at 4× ​magnification.

**Fig. 2:**
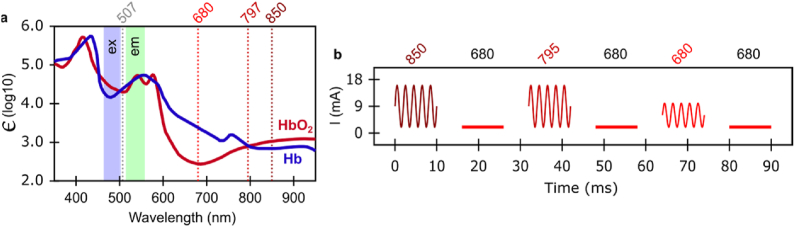
Multi-modality rodent optical imaging system, illumination scheme. **a)** The AF and multi-modal NIR imaging channels compared to hemoglobin molecular extinction coefficient (ϵ). Fluorescent cube ex/em band-pass indicated by blue/green regions, respectively. The dotted red lines show the VCSEL wavelengths. **b)** ​Modality synchronization and current switching sequence for VCSELs.

Similar to Ref. [29], we adapted an Olympus BX61WI microscope for simultaneous back reflectance near-infrared (NIR)-imaging (both laser speckle contrast imaging (LSCI) and intrinsic optical signal imaging (IOSI)) and wide-field fluorescence imaging (see Fig. 1a). A low-magnification air-immersion objective lens (Olympus, Plan N, 4× ​magnification, 0.1 NA) was used to capture a 2×2 ​mm region of the cortex. A multi-wavelength (680 ​nm, 795 ​nm, and 850 ​nm) vertical-cavity surface-emitting laser (VCSEL) package (Vixar, Inc.) was used to provide oblique NIR illumination. The emission wavelength correspondence to the oxyhemoglobin (HbO_2_) and deoxyhemoglobin (HbR) adsorption spectra is shown in Fig. 2a. The NIR and visible light paths were separated with a 660 ​nm long-pass dichroic mirror and filter for simultaneous acquisition. A fluorescein isothiocyanate (FITC) optimized filter cube with a Mercury arc lamp in an Epifluorescence configuration were used for AF imaging (see Fig. 1a and Fig. 2a). The NIR images were recorded at 48 ​Hz (10 ​ms exposure, 2×2 pixel binning) using a QImaging OptiMOS CMOS camera. The AF images were recorded at 1.0 ​Hz (1000 ​ms exposure, 4×4 pixel binning) using a QImaging Rolera EMC^2^ charge-coupled device (CCD) camera. The camera exposure acquisition triggers were used to synchronize the different VCSEL illumination modes, as previously described in Ref. [30]. The VCSEL sources were electrically driven using a Keithley 6221 current source. The individual VCSELs were toggled using a Keithley 7001 electrical current switch system. A driving current profile and source correspondence was repeated every six frames (see Fig. 2b). In every second frame a 680 ​nm VCSEL was driven near threshold, producing a single-mode of coherent illumination suitable for LSCI. In the alternate set of frames, the driving current to the active VCSEL was rapidly modulated producing a sequence of higher-order (lower coherence) modes which result in more uniform ​illumination better suited for IOSI [31]. The central illumination wavelengths of IOSI frames were toggled between 850 ​nm, 795 ​nm and 680 ​nm.

Thermally-pulled glass electrodes with a chloride-coated silver core were used for extracellular potassium and LFP recording. The potassium-selective electrodes (KSEs) were connected to a headstage pre-amplifier (Axon Instruments (AI), CV 203BU) mounted on a micromanipulator (Burleigh, PCS-5000 series). Subsequent analog amplification was performed using an Integrated patch clamp (AI, AxoPatch 200B). The local reference (monopolar ground) for each KSE was the half-barrel of its bound septum electrode. The paired LFP was recorded from the other half of the septum electrode. The LFP channels were each amplified with differential amplifiers (AM Systems, Model 3000 AC/DC). The LFP channels shared a common ground electrode placed in the scalp near the base of the skull. The duplicate potassium and LFP recording channels were aligned roughly perpendicular to one another. The VCSEL light path was oriented roughly perpendicular to one electrode and antiparallel to the other to minimize reflection and lensing of the incident NIR light. The output of each electrical amplifier was recorded using a multi-channel digitizer (AI, DigiData1322A). The acquisition triggers of both cameras were each recorded using the same digitizer to synchronize the imaging modalities with each other and the electrodes (see Fig. 1e). In post-processing, each frame was time stamped using its acquisition trigger midpoint. The sampling rate of all channels was 10 ​kHz, enabling 100 μs timing precision in modality correlation. An active digital high-pass filter with cut-off frequency 0.1 ​Hz was applied to the LFP, particularly during two channel recordings (for live comparison). An active analog low-pass filter with cut-off frequency of 5 ​kHz was applied by the KSE amplifiers for suppression of aliasing.

### Potassium electrode fabrication

The procedure to manufacture KSEs was similar to previous studies [1,[32], [33], [34], [35]]. In brief, the functionalized potassium electrodes were made from glass microelectrodes (∅1.50 ​mm outer, ∅1.12 ​mm inner, World Precision Instruments TW150F-4). Electrode barrels were pulled using the Narishige puller with approximately 2–5 ​μm diameter openings for both the LFP reference and KSEs.

Glass scintillation vials containing dimethylchlorosilane (DMCS) and ethanol were prepared under a fume hood. The liquid level was maintained at approximately 5 ​mm, covering the bottom of the respective vials. The DMCS vial was wrapped in parafilm during storage. The ion-sensitive electrodes were placed in a holder-cap (a scintillation vial cap with holes drilled for electrodes) such that the electrodes did not protrude below the bottom of the cap. The electrodes were exposed to the DMCS vial for 50 ​s and then to the ethanol vial for 50 ​s. Electrodes were placed in a cardboard holder and baked at 80 ​°C for 2 ​h. Electrodes were stored in a desiccator for no more than 6 weeks.

Electrode silver wires were flame-cleaned to remove the old coating, bleached for 30 ​min and rinsed in ddH_2_O. There are two head-stage leads, four double barrel leads and one ground electrode. Filling and binding of electrodes was done within 1 ​h of the experiment. With a Hamilton syringe (or melted syringe) the tip of the ion-sensitive barrel was filled with 0.3 ​μL of ion-sensitive gel (potassium ionophore I-cocktail B). Bubbles were removed by flicking the barrel, using external pressure, or dragging with a thin plastic filament. The tip of the electrode was placed downward for 5 ​min permitting the gel to move down with gravity. The electrodes were back-filled with 200 ​mM KCl. The reference barrel and LFP were filled with PBS.

Paired electrodes were built by bonding septum glass electrodes with silanized KSEs. Electrodes were placed in a spring clamp with soft plastic swivelling jaws which enabled precise tip-pair alignment prior to barrel bonding. Barrels were bonded with rigid packing tape pre-cut to ascend the tapered barrel alignment. Electrodes were calibrated by placing them in artificial cerebrospinal fluid (ACSF) solutions with different [K^+^]: 0.3, 3.0, and 30 ​mM. The ACSF was prepared with 500 ​mL ddH2O (in mg): 3500 NaCl, 900 Glucose, 246 MgSO_4_, 86 NaH_2_PO_4_, 925 NaHCO_3_, and 147 CaCl_2_. The pH was adjusted to 7.4 prior to addition of KCl.

### *In vivo* surgical procedure

All procedures were approved by the animal care committee at the Krembil Research Institute, University Health Network. All experiments were conducted on one-to-four month old CD-1 mice (mass 20–35 ​g, *n* ​= ​40). Mice were anesthetized in an induction chamber using 5 % isoflurane in 30 % O_2_/N_2_ (0.8 ​L/min). After induction the mouse was transferred to a nose cone with ear bars. The animal was maintained on a heating pad at 37 ​°C with rectal thermometer regulation (Harvard Apparatus). A craniotomy was performed leaving the dura layer intact. Chilled PBS was used to reduce heat from the drill. The exposed dura was covered with a piece of SurgiFoam soaked in PBS. The animal was then directly transferred (and secured) to the imaging platform. Isofluorane was maintained at 2 % from this point forward until the end of data acquisition.

### Recording procedure and induction of SDs

Two sets of multi-barrel electrodes (paired KSEs and standard micro-electrodes) were inserted into the cortex through the dura. A single glass capillary filled with 0.5 ​M KCl was inserted into the cortex for SD induction (see Fig. 1b). The multi-barrel electrode bundles focal and distal to the KCl injection site were placed ∼ 2 ​mm away from each other. The capillary was placed within ∼ 100–300 ​μm of the focal electrode bundle. All electrodes and the glass capillary were inserted to a depth of 400–600 ​μm below the cortical surface. Individual SD events were induced by applying 8 ​ms pressure pulses to the glass capillary (Picospritzer II, 40 psi). The oblique multi-wavelength VCSEL package was aligned to provide NIR illumination for blood flow and tissue oxygenation imaging. The [K^+^]_e_ was measured using our previously shown approach [1], enabling concurrent recording of the LFP associated with the potassium reference potential. A minimum induction interval of 4 ​min was chosen based on the ability to reliably produce repeatable SD events and gain sufficient samples while limiting time under anesthesia. If the observed pre-drug administration [K^+^]_e_ waveforms were longer, the minimum interval was extended for that experiment. As there are longer lasting physiological effects associated with SD, we do not assert that each repeated induction reflects a start from a consistent baseline amongst all physiological parameters.

### Drug administration and euthanasia

Prior to surgery, MQH (Sigma-aldrich) was dissolved in DMSO at 20 ​mg/mL (solubility 38 ​mg/mL) and diluted by 100× ​in PBS prior to intraperitoneal (IP) injection. Alternately, MFA (Sigma-aldrich) was dissolved in water at 50 ​mg/mL and diluted by 167× ​in PBS prior to injection. Mice were weighed prior to surgery to calculate drug dosage. Both drugs were administered at 1 ​mg/kg via IP injection after the recording of baseline SD events (maximum volume 0.2 ​mL). Mice were euthanized using pentobarbital (100 ​mg/kg, IP injection).

### Analysis

The relative blood flow was calculated for LSCI using the speckle flow index (SFI) (as in Ref. [30]). The Δ[HbR], Δ[HbO_2_], and Δ[HbT] for IOSI were computed from the relative imaging intensities at *R*_680_, *R*_795_, and *R*_850_ (as in Ref. [30]). During post-processing, regions of interest were selected based on the optimal wavefront signal-to-noise ratio (SNR). Twenty-four frame mean binning was applied to the LSCI and IOSI traces shown. Parameters were extracted from LFP and [K^+^]_e_ traces for statistical comparison. A digital high-pass filter of 0.001 ​Hz was applied to all channels. We report the quantification of individual experimental procedures as percentage change between the mean of pre- and post-drug administration single SD event quantifications. All hypotheses were assessed with two-tailed one-sample t-tests applied to groups of percentage change values associated with a given drug. Error bars indicate mean ​± ​s.e.m. in all graphs.

### Optical wavefront localization for mapping SD dynamics

The AF images were first temporally normalized (pixel-wise) using a rolling normalization filter with a 10 s reference window. The normalized imaging series has a reduced vessel appearance but is noisy compared to the amplitude of the SD signal. A bivariate standardized moment filter [36] was applied to each normalized image. Using a circular filter kernel of sufficiently large radius enables detection of the leading edge of the SD wave. Interleaved maps from the filters were used for visual estimation of SD spatial location of onset. The wavefront maps were angularly-projected to merge wavefront radial-location estimates from across the full field of view (FOV). Smaller diameter bivariate standardized moment filters were used to identify false positive SD features. Separately, image displacement for tissue tension tracking was assessed using the histogram kurtosis of the blood flow map temporal derivative.

### Potassium wavefront localization for SD event synchronization

The temporal leading edge of potassium waves were marked using a rolling univariate standardized moment filter. These were chosen as they demonstrated low noise and negligible sensitivity to filter length. As injection volumes required for induction were variable, this approach provided an unbiased option for common time alignment of depolarization events.

### Azimuthal parametrization scheme

The principle of operation for the azimuthal wavefront parametrization scheme introduced in this work is shown in Fig. S1. The corresponding mapping principle can be applied to a fixed-rate expanding circle, where a parametrization filter (also bivariate moment-derived) moves along the circle corresponding to each time-point. Relative to the expanding circle, deviation in the propagating wavefront can be mapped by the parametrization filters. Angular projection of wavefront localization maps over time permits the visualization of radial velocity profiles (see Fig. S1a for filter con). For more variable velocity wavefronts or drifting origin wavefronts (see Fig. S1b later time point) circle realignment prior to azimuthal parameter extraction is required (see Fig. S1c) before subsequentmapping. The two most direct approaches and their corresponding circle fitting algorithms are described below.

The simplest approach is least-squares fitting of radius for a fixed centre. Using the implicit equation of a circle, *r*^2^ = (*x* ​− ​*a*)^2^ ​+ ​(*y* ​− ​*b*)^2^, and using the substitution to a predefined fixed center, *u* ​= ​*x* ​− ​*a*, and, *v* ​= ​*y*−*b*, the least-squares fit can be trivially computed from expectation values, $\left\langle r^{2}\right\rangle =\left\langle u^{2}\right\rangle +\left\langle v^{2}\right\rangle$, where, $r_{fit}=\sqrt{\left\langle r^{2}\right\rangle }$, Parameters extracted along the azimuthal region spanning this radius can be mapped back to spatial coordinates (see Fig. S2a).

The next most direct approach is a complete least-squares circle fit. Starting from a circle refactored into a linear equation [37]

*r*^2^ = (*x* ​− ​*a*)^2^ ​+ ​(*y* ​− ​*b*)^2^

(*r*^2^ ​− ​*a*^2^ ​− ​*b*^2^) ​+ ​(2*a*) *x* ​+ ​(2*b*) *y* ​= ​*x*^2^ ​+ ​*y*^2^

*c*_0_ ​+ ​*c*_1_*x* ​+ ​*c*_2_*y* ​= ​*d*

We have a corresponding overdetermined system, *A***c** ​≈ ​**d**, where

$$A=\left(\begin{array}{ccc} 1 & x_{1} & y_{1}\\ \vdots & \vdots & \vdots \\ 1 & x_{n} & y_{n} \end{array}\right),\;c=\left(\begin{array}{c} c_{0}\\ c_{1}\\ c_{2} \end{array}\right),\;d=\left(\begin{array}{c} d_{1}\\ \vdots \\ d_{n} \end{array}\right)$$

After solving the linear system (*A*^⊤^*A*)**c** ​= (*A*^⊤^**d**), we get the conventional circle parameters by substitution; $a=\frac{c\_1}{2}$, $b=\frac{c\_2}{2}$, and *r*^2^ ​= ​*c*_0_ ​+ ​*a*^2^ ​+ ​*b*^2^. For a weighted least squares solution, the weighted matrix can be computed from second-order moments

$$A^{⊤}WA=\sum_{x,y}\left(\begin{array}{ccc} 1 & x & y\\ x & x^{2} & xy\\ y & xy & y^{2} \end{array}\right)w\left(x,y\right)$$

and the weighted vector from third-order moments

$$A^{⊤}Wd=\sum_{x,y}\left(x^{2}+y^{2}\right)\left(\begin{array}{c} 1\\ x\\ y \end{array}\right)w\left(x,y\right)=\sum_{x,y}\left(\begin{array}{c} x^{2}+y^{2}\\ x^{3}+y^{2}x\\ y^{3}+x^{2}y \end{array}\right)w\left(x,y\right)$$

Which provides a weighted solution for **c** from (*A*^⊤^*WA*)**c** = (*A*^⊤^*W***d**) where *W* is a diagonal matrix of weights *w*_*ii*_ ​= ​*w*(*x*_*i*_*,* *y*_*i*_). In this work, weights are the values from our optical wavefront localization filter. Extracted parameters can similarly be mapped to spatial coordinates (see Fig. S2b).

## Results

### Near complete suppression of SDs after MFA-administration

**Fig. 3:**
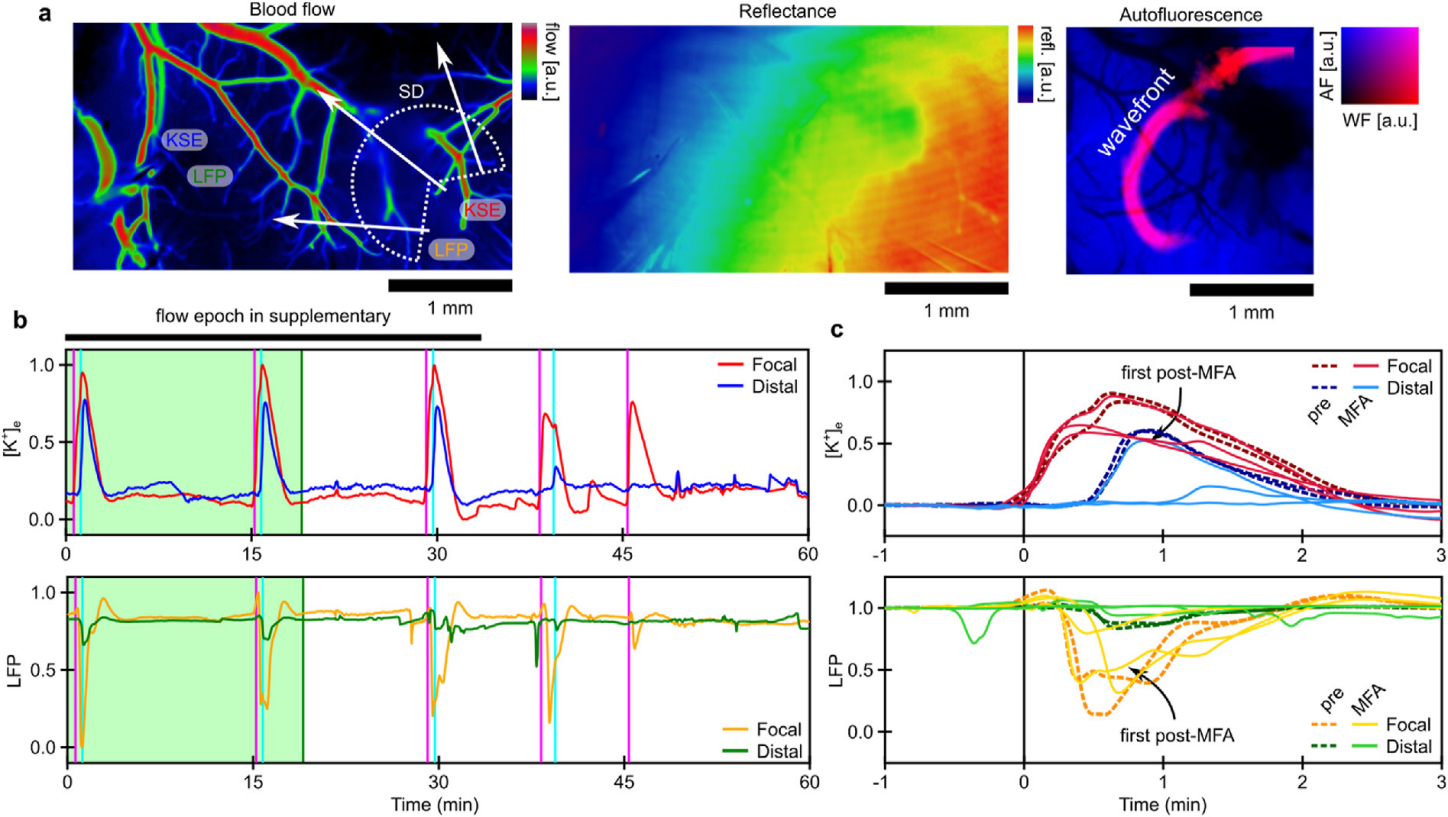
Suppression of SD with acute MFA administration (individual example). **a)** Blood flow map from LSCI, with IOSI and AF maps acquired concurrently. The LFP and KSE locations are indicated (label colors correspond to panel b traces). **b)** Recording of [K^+^]_e_ and LFP both adjacent and distal to the sight of SD induction (shaded green epoch is before MFA administration). Vertical lines indicate SD onset as classified by the individual [K^+^]_e_ recordings (adjacent/magenta and distal/cyan). **c)** ​Event onset overlaid traces synchronized to onset as defined by adjacent potassium trace (dashed traces represent pre-MFA administration).

**Fig. 4:**
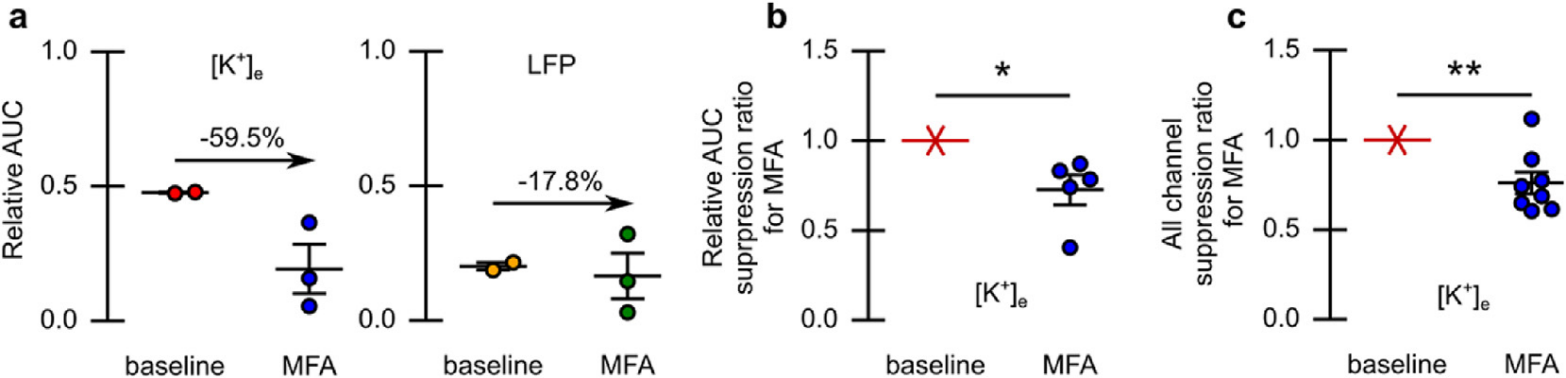
Suppression of SD with acute MFA administration. **a)** Treatment-induced change in the relative AUC between focal and distal [K^+^]_e_ and LFP recording (for example in Fig. 3). Change in relative AUC post-MFA administration indicated. **b)** Treatment-induced change in the relative AUC between focal and distal [K^+^]_e_ for all animals with stable channel dual [K^+^]_e_ recording (n ​= ​5). **c)** Treatment-induced change in the AUC of [K^+^]_e_ for all animals (n ​= ​8). Error indicated by mean ​± ​s.e.m. ∗*P* ​≤ ​0.05, ∗∗*P* ​≤ ​0.01.

To assess the impact of inter-astrocytic communication blockade on SD dynamics, we conducted multimodality monitoring of tissue dynamics before and after MFA administration. MFA was administered via IP injection (1 ​mg/kg) after control event induction. Blood flow and tissue reflectance were mapped over a cortical region concurrently with electrographic recording (see Fig. 3a). The relative location of electrodes is indicated on the blood flow map. The SD propagating wavefront is shown atop a map of tissue AF (from which it was computed using our enhanced wavefront detection scheme). The SD events following MFA administration began to experience a reduction in both the focal and distal potassium and local field components of SD (see Fig. 3b). Overlaying the SD events, based on the detected onset-time, permits the quantification of this reduction through the area under curve (AUC) for the potassium trace (see Fig. 3c). Distal [K^+^]_e_ signals were reduced relative to focal [K^+^]_e_ signals as assessed through the AUC ratio for each event (see Fig. 4a). This reduction (−27±8 ​%; *p* ​= ​0.03, two-tailed one-sample *t*-test, n ​= ​5 mice) was consistent across all MFA experiments with stable dual channel [K^+^]_e_ recordings (see Fig. 4b). Across all experiments a general reduction (−24±6 ​%; *p* ​= ​0.006, two-tailed one-sample *t*-test, n ​= ​8 mice) in AUC of the [K^+^]_e_ signal for SD-events was observed (see Fig. 4c). In this example, the SD event directly following MFA administration experienced reduced blood flow distal to the SD induction site (see Fig. S3).

The relative reduction in distal compared to focal [K^+^]_e_ signals was observed when SD-induction was not completely suppressed (see Fig. S4). Another representative example of MFA-induced suppression is shown in Fig. S5. In this example, there was again a reduction in observed susceptibility to SD induction, as quantified by a reduced AUC for the focal electrode. Similar reductions in SD susceptibility were consistent across experiments (see experiment summaries in Fig. S6a-e). In most cases, changes in the LFP component of SD events mirrored the [K^+^]_e_ component. However, trends due to MFA administration were not consistent in the LFP data.

### Delay and intermittent suppression of SDs after MQH-administration

**Fig. 5:**
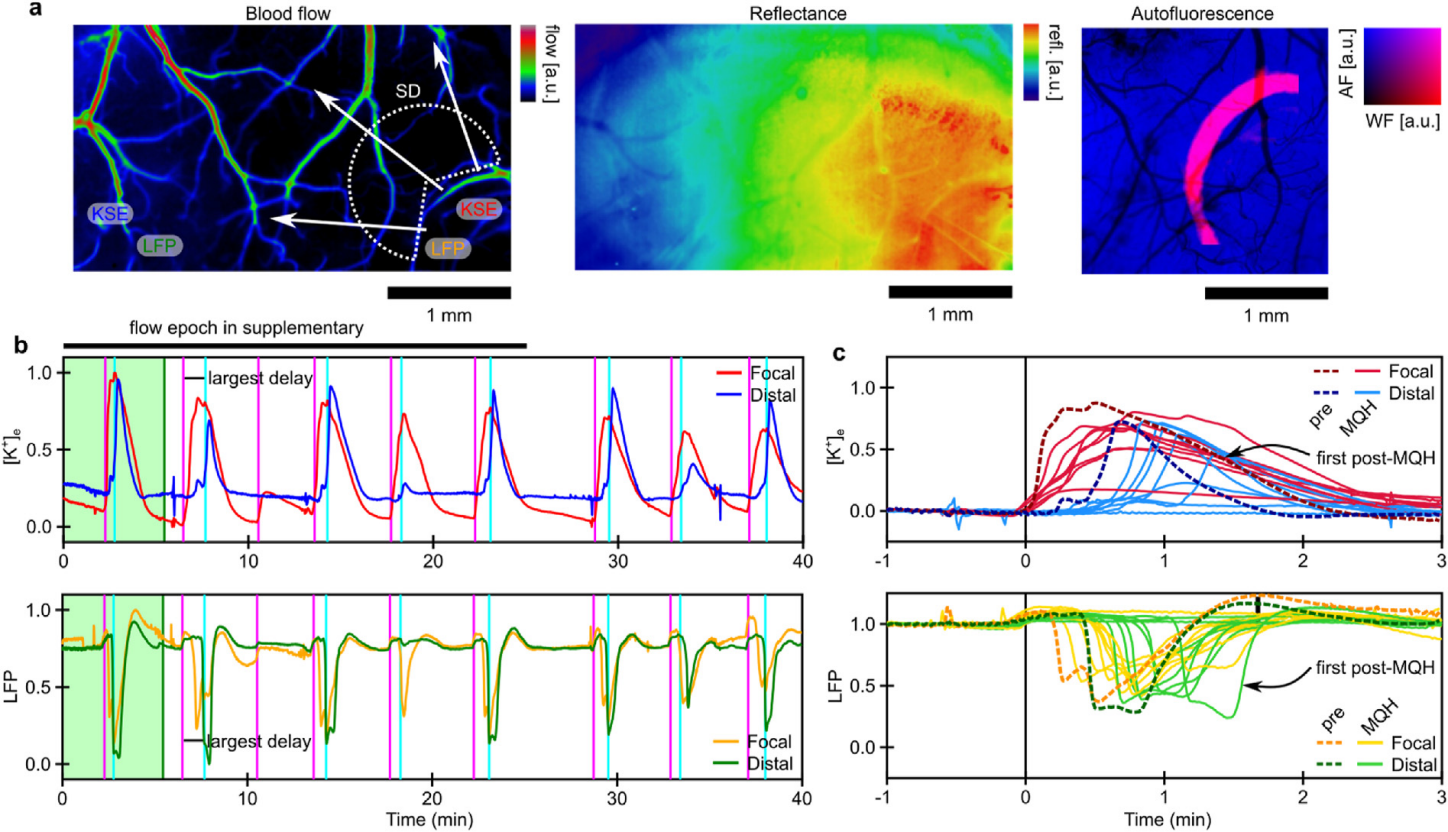
Modulation of SD propagation with acute MQH administration (individual example). **a)** Blood flow map from laser speckle flowmetry, with IOSI and AF map acquired concurrently. Electrode locations are indicated with their corresponding traces in panel-b indicated by color. **b)** Recording of [K^+^]_e_ and LFP both adjacent and distal to the site of depolarization induction (shaded green epoch is before MQH administration). Vertical lines indicate the onset of events as classified by the individual [K^+^]_e_ recordings (adjacent/magenta and distal/cyan). **c)** Overlaid SD traces synchronized to onset as defined by induction-adjacent potassium trace (dashed traces are before MQH administration). Distal recordings from the first post-MQH event are indicated as it had the most significant delay from the focal response.

**Fig. 6:**
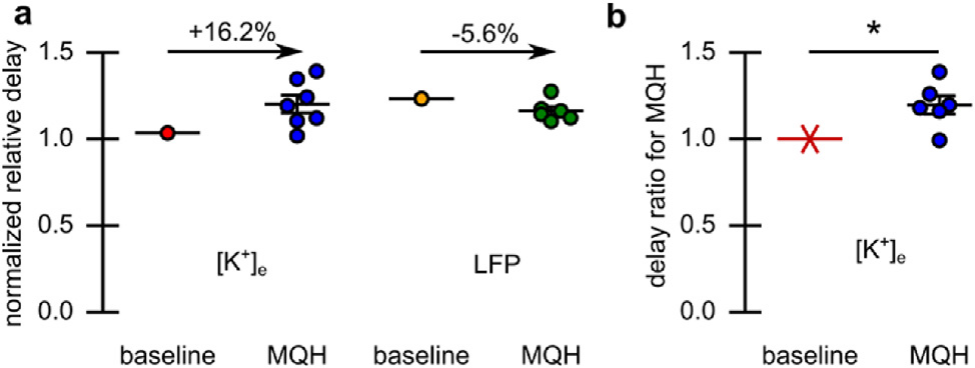
Modulation of SD propagation with acute MQH administration. **a)** ​Treatment-induced change in the relative distal-to-focal event timing for both the [K^+^]_e_ and LFP recordings for the experiment in Fig. 5 and **b)** for the [K^+^]_e_ recordings for all MQH-experiments (n ​= ​6 mice). Error indicated by mean ​± ​s.e.m. ∗*P* ​≤ ​0.05.

To assess the impact of blocking both inter-astrocytic and inter-neuronal communication on SD dynamics, we conducted multi-modality monitoring of tissue dynamics before and after MQH-administration. A dose of 1 ​mg/kg MQH was administered via IP injection acutely after induction of baseline events. An exemplar map of blood flow over the entire cortical FOV is shown in Fig. 5a. The relative placement of electrodes and dynamics of SD propagation are indicated. The IOSI and AF channel are shown to the right. In this example, some events following MQH administration had no distal blood flow response (see Fig. S7). Several events directly following MQH administration were reduced focally and distally with respect to the potassium and local field components of SD (see Fig. 5b). The consistency of event amplitude reduction was less than for MFA. However, common time events showed an increase in the delay from focal to distal [K^+^]_e_ responses and small decreases in field potential responses (see Fig. 5c). The ratio of focal to distal event mid-point (temporal expectation value) was used to quantify this change in region response timing (see Fig. 6a). The time delay associated with [K^+^]_e_ (+19.7±5 ​%; *p* ​= ​0.01, two-tailed one-sample *t*-test, n ​= ​6 mice) was observed consistently after MQH administration (see Fig. 6b). Additional representative examples of MQH-induced intermittent SD suppression and delay of SD propagation are shown in Fig. S8 and Fig. S9. This delayed SD propagation was consistent with three further experiments (see summaries in Fig. S10a-c).

Delayed SD propagation is a somewhat expected result for the blocking of gap junctions. One potential cause is that SD associated astrocytic [Ca^++^]_i_ waves can no longer proceed through fast interastrocytic gap junctional communication. This assertion is supported by the fact that the abolishment of [Ca^++^]_i_ waves slows SD-onset [38]. However, in our data the initial [K^+^]_e_ propagation delay was in instances reduced over time indicating other factors, such as adenosine triphosphate (ATP) extrusion, are likely involved in the observed delay variability. However, MQH inhibits ATP-sensitive potassium-channels [39]; specifically, Kir6.2 which is expressed in neurons but not astrocytes [40]. Therefore, suppressed astrocytic gap junctional communication is likely not the only factor limiting the rate of SD propagation.

### Treatment-induced changes in SD-associated blood flow response

**Fig. 7:**
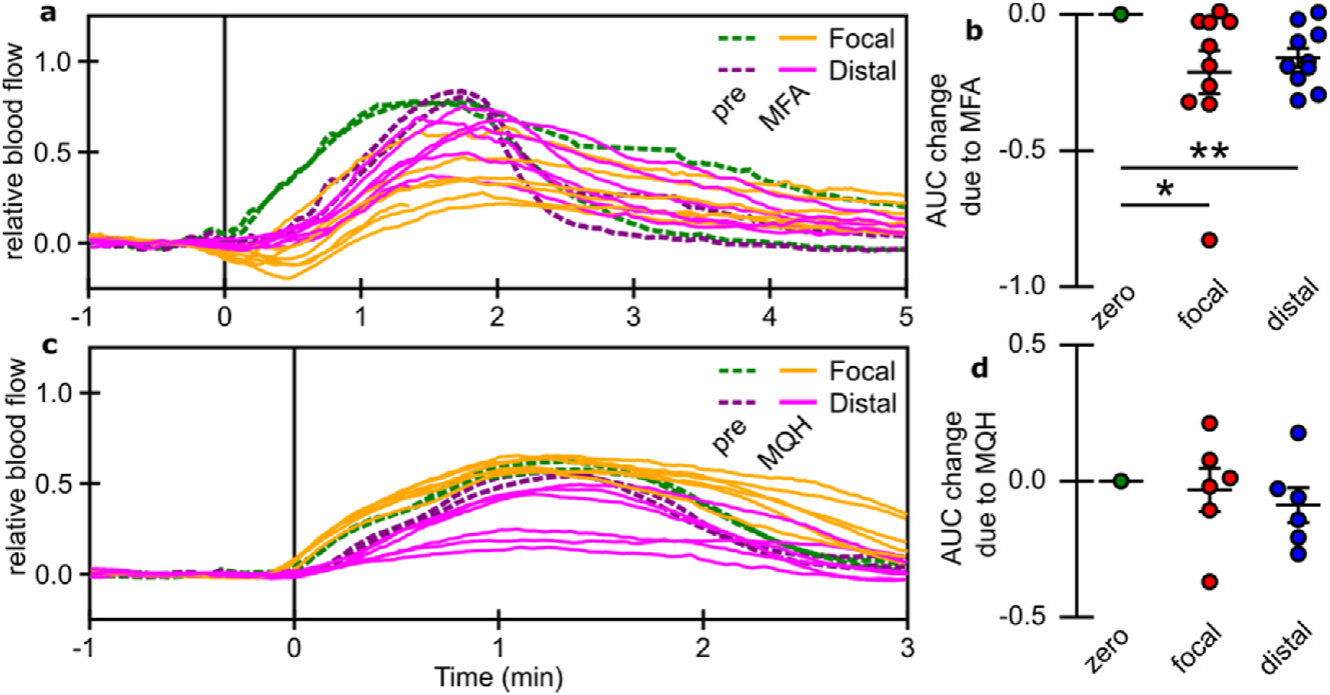
Relative blood flow synchronized to potassium SD-wave onset. **a)** Blood flow before and after MFA administration in both focal and distal regions of the cortex. **c)** Blood flow before and after MQH administration in both focal and distal regions of the cortex. **b,d)** Summary across experiments of the average AUC change due to the administration of MFA (n ​= ​10) and MQH (n ​= ​6), respectively, for both focal and distal regions of the cortex. Error indicated by mean ​± ​s.e.m. ∗*P* ​≤ ​0.05, ∗∗*P* ​≤ ​0.01.

Concurrent blood flow mapping was used to assess the hemodynamics response concomitant with changes in electrographic activity. The administration of MFA produced changes in SD-induced cerebral blood flow similar to those observed for [K^+^]_e_ in the majority of cases (see Fig. 7a and Fig. S11a-g). The change was most marked during the SD-onset. These changes were quantified using the change in flow AUC prior to peak flow (see Fig. 7b). For both focal and distal regions, SD-induced blood flow changes were reduced at onset. There were also delays in the SD-induced blood flow return to baseline. Conversely, the administration of MQH did not produce consistent changes in blood flow (see Fig. 7c and Fig. S12a-e). Quantification with change in AUC reflects the variability in blood flow response (see Fig. 7d). Variability in blood flow wavefronts precludes precise analysis of event temporal expectation (as had been done for [K^+^]_e_ traces). However, a slowing of ​return to baseline appears to be observed after MQH administration.

We note, as our imaging acquisition system maps SD dynamics independent of stable electrographic recording, the flowmetry analysis was possible on a few experiments which lacked reliable electrographic data. For the majority of cases both blood flow and [K^+^]_e_ could be analyzed together. Despite an observable degree of similarity in SD-associated dynamics, a robust dynamical relationship was not observed between blood flow and [K^+^]_e_ recordings, as assessed through phase-space correlation analysis (data not shown).

### Occurrence of regional differences in SD tissue oxygenation

**Fig. 8:**
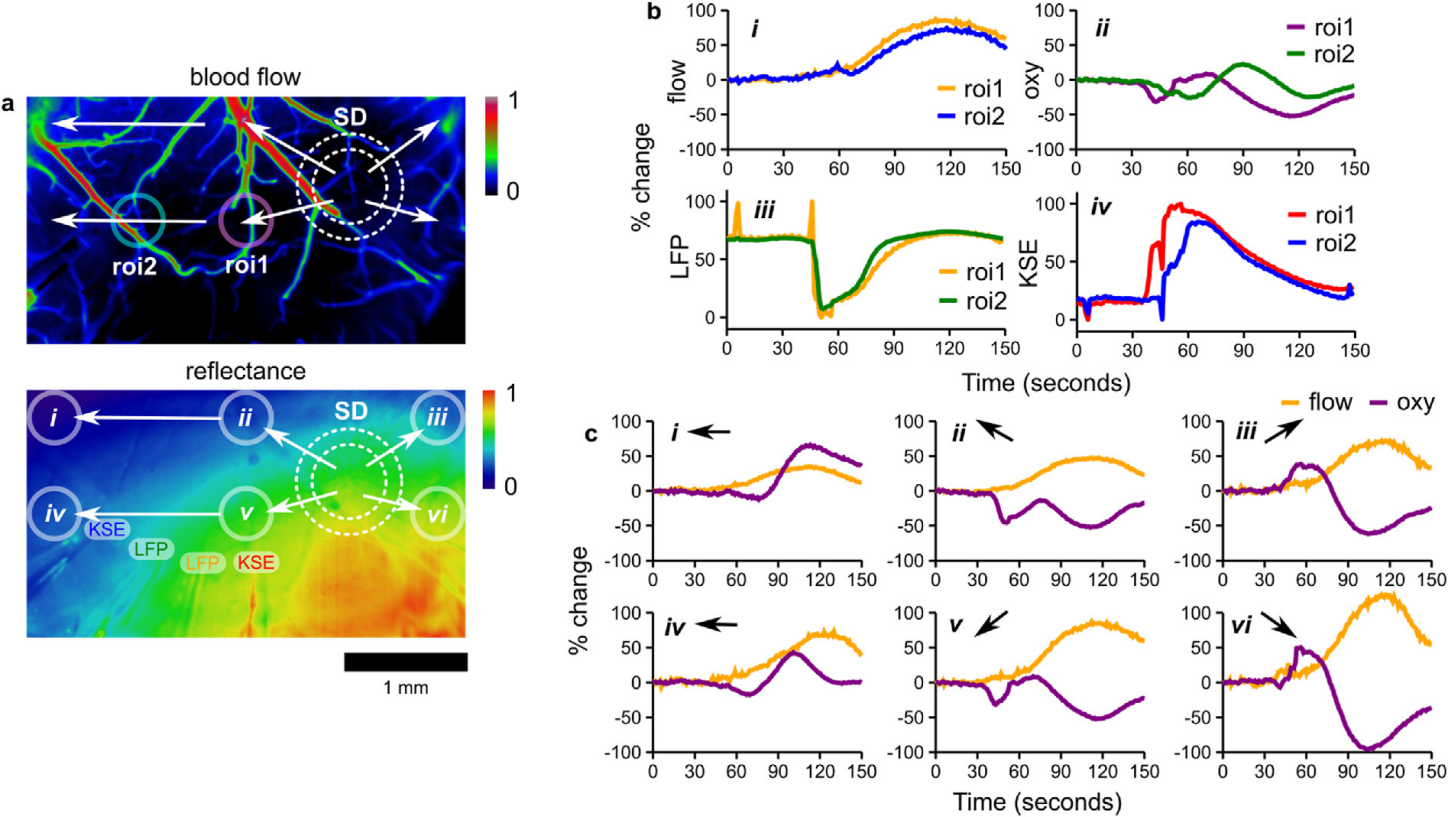
Variability in tissue oxygenation despite qualitatively consistent blood flow. **a)** Blood flow (top) and tissue reflectance (bottom) maps prior to SD with superimposed ROIs for temporal traces. Arrows and dashed white circles indicate SD propagation direction. **b)** Temporal traces of *i)* blood flow and *ii)* tissue oxygenation for the two ROIs proximal to the paired *iii)* LFP and *iv)* KSE tips with their corresponding traces. **c)** Temporal traces of blood flow versus tissue oxygenation for ROIs across the FOV labeled *i-vi* in reflectance map.

Concurrent oxygenation mapping was used to assess tissue hypoxia and fast optical dynamics associated with SD. Several instances of regional blood flow response variation were observed. In such instances, distinct arterioles were responsible for most of the variation, often appearing to reflect alternate routes of SD propagation that lead or lag the propagation within the FOV. However, blood flow temporal dynamics were typically highly similar within the FOV and propagated in a radial manner with a broad wavefront (see Fig. 8a). Despite observed blood flow uniformity, tissue oxygenation dynamics could vary independent of blood flow (see Fig. 8b). Firstly, tissue oxygenation was more likely to reflect the propagating nature of SD, with clear time delays in adjacent regions of interest (see Fig. 8b *ii* versus *i*). This more closely reflects delays between KSE recordings but not LFP at this scale (see Fig. 8b *iv* versus *iii*). Secondly, tissue oxygenation could involve an initial dip at SD onset in some regions (see Fig. 8c *ii* and *v*) not seen across the whole FOV (see Fig. 8c *i*, *iii*, *iv*, and *vi*). Lastly, some regions experience a large rebounding reflectance change during peak blood flow (see Fig. 8c *ii*, *iii*, *v*, and *vi*) while others simply returned directly to baseline (see Fig. 8c *i* and *iv*).

### Reduction in tissue tension with MQH

**Fig. 9:**
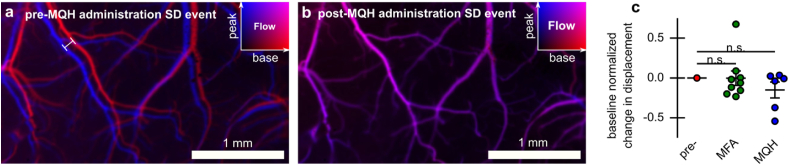
Reduction in SD-wave induced tissue displacement after MQH administration. **a)** Superimposed blood flow maps from just before SD-wave (red) and at the peak of the SD-wave (blue) occurring. Maximum translation of tissue due to tension indicated by the line segment. **b)** Same as panel-a for SD event occurring after MQH administration. **c)** Change in displacement due to MFA and MQH administration based on event integrated movement parameter. Error indicated by mean ​± ​s.e.m.

SDs involve significant changes in vascular tone leading to regional variation in vasodilation and constriction [[41], [42], [43]]. These changes in vascular tone for global events like SD and seizures, in principle, can add to large local displacements of cortical tissue. Moreover, this can be potentially exacerbated by known neural cell swelling [21]. Through high-contrast blood flow mapping this was often observed in our experiments (see Fig. 9a). Interestingly, after MQH administration, the SD-induced tissue displacement was substantially reduced as shown in Fig. 9b. This reduction pressure/tension could alleviate a source of SD-induced damage not typically targeted. In cases where baseline events did not show displacement there was no change in response. Using a measure of image displacement integrated over event epochs, neither MFA or MQH significantly affected events across cases (see Fig. 9c). This is likely due to cases where baseline SD events have little associated movement. In these cases, the sensitivity for our chosen movement parameter may also be insufficient.

### Mapping SD dynamics with wavefront localization in low-contrast epifluorescence and reflectance images

**Fig. 10:**
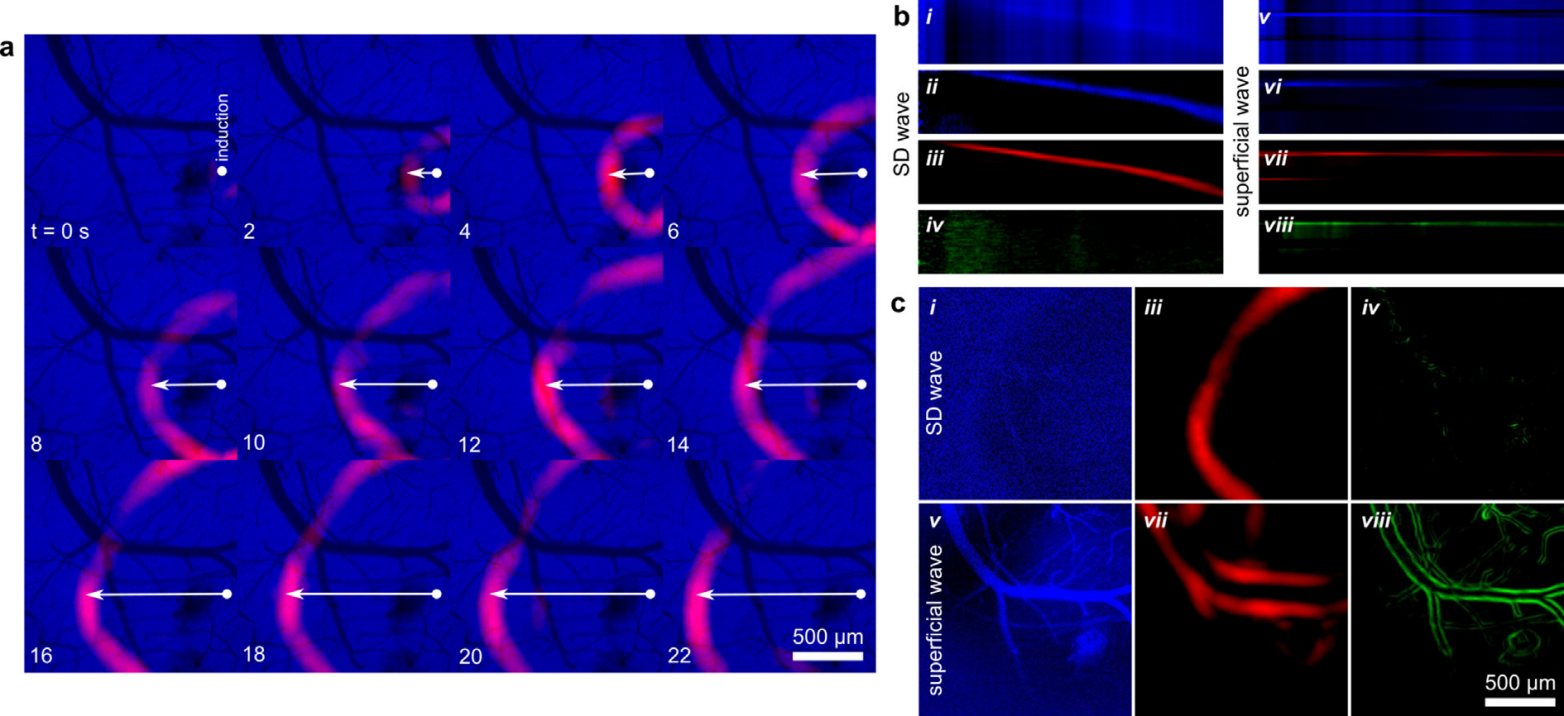
Full-field SD wavefront isolation from low-contrast AF images. **a)** Computed wavefront (red) superimposed on source images (blue). Spatial bivariate standardized moment filters were applied after temporal normalization filtering. **b)** SD-event from angularly-projected view for *i)* the AF images, *ii)* the AF images with post-projection de-striping, *iii)* the computed SD-wavefront images, and *iv)* vessel-scale computed wavefront images. *v-viii)* Same as *i-iv* for a distinct wavefront due to superficial fluid motion. **c)** Select time points from panel-b (same *i-viii* correspondence).

We sought to track changes in wavefront propagation dynamics associated with SD. Firstly, the SD wavefronts were most sharply localized in our AF imaging channel (see Fig. 10a), enabling the precise spatial localization of the SD-onset with respect to the cortical surface. Angular projection of parameter maps around this location enables precise tracking of SD wavefront dynamics (see radial-temporal profiles in Fig. 10b). By taking the unfiltered AF signal projection (see Fig. 10b *i*) and applying destriping (horizontal and vertical) we see a well defined SD wavefront (see Fig. 10b *ii*). This wavefront closely matches the projected filter wavefront (see Fig. 10b *iii*) but for a short phase delay. Consequently, our filtering approach can spatially map out a denoised variant of the low-contrast AF intensity spike.

For much smaller filter diameters (on the order of vessel edges), the SD wavefront is essentially invisible (see Fig. 10b *iv*). This allows us to discriminate our false positive SD-like signals, such as superficial fluid flow (see Fig. 10b *v*-*viii*) or tissue motions, as these contribute to the small filter signal. To clarify, high-noise temporal-gradients (see Fig. 10c *i*) are used to derive the SD wavefront map (see Fig. 10c *iii*). Other events create gradients (see Fig. 10c *v*), which are similarly detected by our filter (see Fig. 10c *vii*). However, these other events are distinct from SD when using smaller filters maps (see Fig. 10c *iv, viii*). Consequently, we can precisely classify SD events spatiotemporally.

**Fig. 11:**
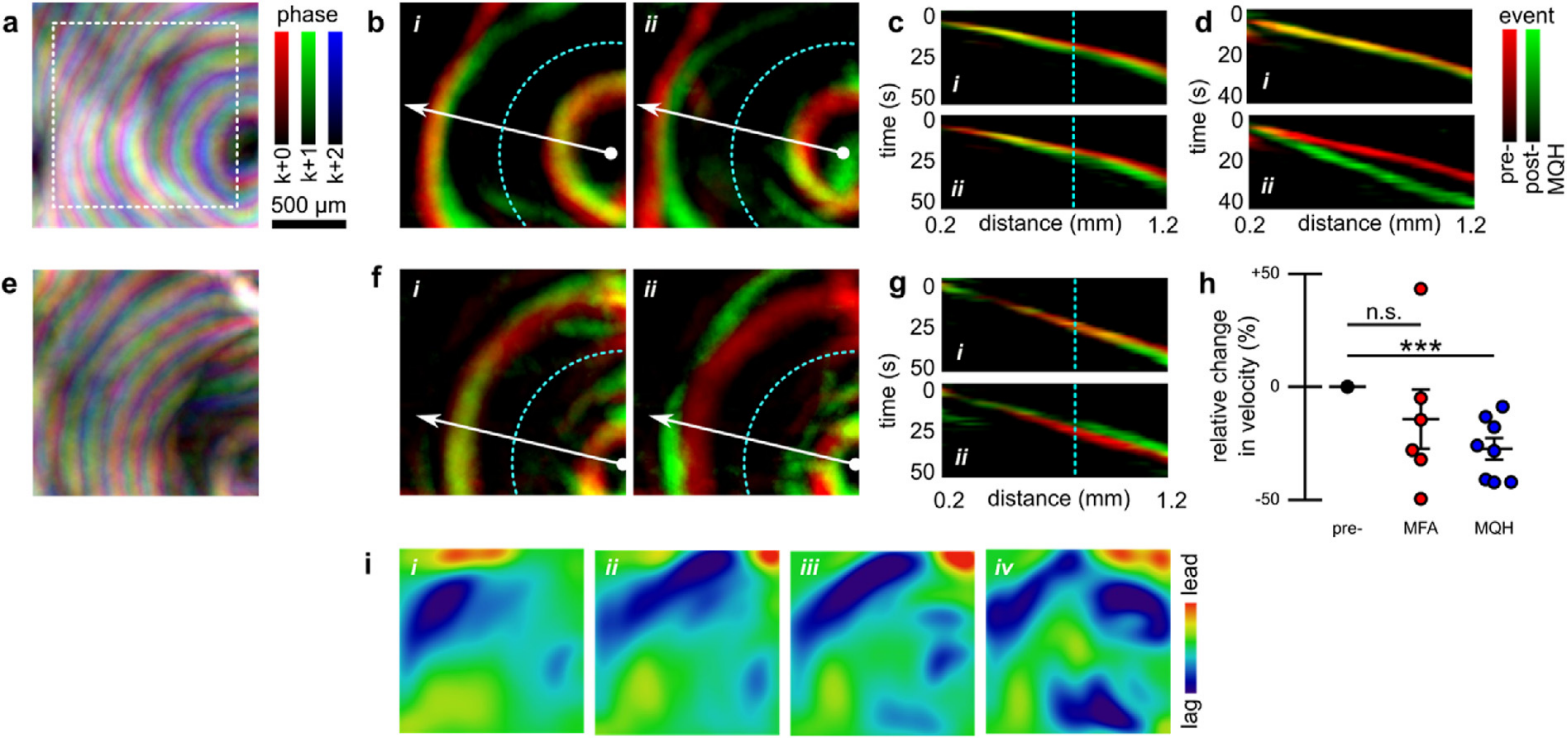
Measuring changes in SD wave velocity from computed SD wavefronts. **a)** Map of wavefront propagation through interleaved time-point superposition highlighting spatial variation in propagation. Three relative phase delays for map time-points at *k*, *k*+1, and *k*+2 ​s (for *k* ​= ​3*n* and *n* ​∈ ​N) are shown in red, green, and blue, respectively. **b)** Superimposed 20s offset wavefront maps from before (red) and after (green) MQH administration, capturing the reduction in SD in velocity. The delay increased between the *i)* first and *ii)* second post-MQH events. **c)** Angularly-projected view of the SD events corresponding to the maps shown in panel-b. The vertical line (cyan) indicates location corresponding to semi-circle (cyan) in panel-b. **d)** Same as panel-c for MQH experiment with progression to large wavefront delay. **e-g)** Same as panels a–c but corresponding to MFA administration, which both *i)* delayed and *ii)* hastened SD propagation. **h)** Summary statistics for relative change in mean event velocity for all MFA and MQH experiments with sufficient resolvable wavefronts. **i)** Deviation of wavefront from circular propagation assessed with azimuthal parametrization scheme. Output corresponds to inset region in panel-a for four sequential SDs, the first being the pre-MQH event shown above. Error indicated by mean ​± ​s.e.m. ∗∗∗*P* ​≤ ​0.001.

The SD wavefront localization scheme allows precise mapping of spatial variation in SD dynamics (see Fig. 11a). From these mappings we can compare SD propagation dynamics before and after MQH administration (see Fig. 11b). Our results show that optical correlates of the SD wavefront were delayed by MQH administration. The change in wavefront velocity was quantified using the slope of the angularly-projected filter maps (see radial-temporal profiles in Fig. 11c). An example showing the development of a larger MQH-induced wavefront delay is shown in Fig. 11d. After MFA administration, SD wavefront velocity was typically reduced. However, the velocity reduction was less consistent. For example, post MFA administration SD events both slower and faster than baseline could be observed (see Fig. 11e–g). For all SD events with sufficiently resolvable wavefronts, MQH administration was found to significantly reduce velocity (Δ*v/v*_0_ ​= ​−27±5 ​%, mean ​± ​s.e.m.; *p* ​= ​0.0007, two-tailed one-sample *t*-test; Cohen's *d* ​= ​−2.04), whereas MFA administration did not produce a significant reduction (Δ*v/v*_0_ ​= ​−14±13 ​%, mean ​± ​s.e.m.; p ​= ​0.32, two-tailed one-sample *t*-test; Cohen's *d* ​= ​−0.45) (see Fig. 11h). Our measured baseline velocities (2.7±0.2 ​mm/min, mean ​± ​s.e.m) were consistent with the literature and, as expected, were similar between groups (*v*_*MFA*_ ​− ​*v*_*MQH*_ ​= ​0.2±0.5 ​mm/min, difference of means ​± ​propagated s.e.m. for linear combination; *p* ​= ​0.66, two-tailed two-sample *t*-test; Cohen's *d* ​= ​−0.28).

Each wavefront from the temporal projection in Fig. 11a can be separately aligned with a least-squares circle of best fit. This permits arbitrary bivariate moment-derived characterization using our azimuthal parametrization algorithm described in section 2.9 (see Fig. S1). Here we show the spatial parameter mapping of wavefront lead-lag compared to drifting circular propagation (see Fig. 11i). The algorithm and limitations of the different initialization approaches are discussed in section 2.9, where simulated wavefronts are used to emphasize the differences (see Fig. S2).

**Fig. 12:**
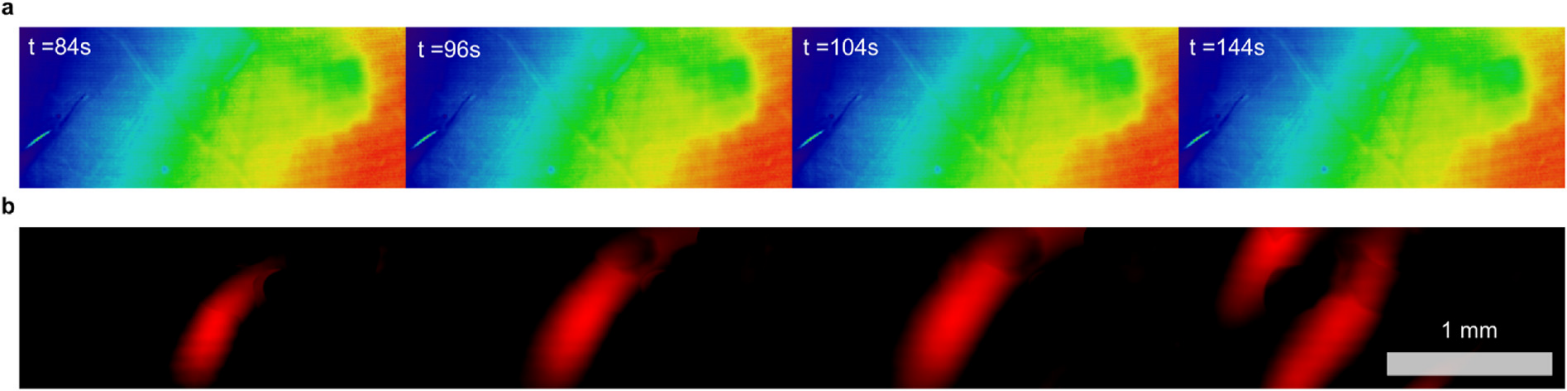
Enhanced SD wavefront computation from low-contrast intrinsic optical signal images. This approach applies spatial bivariate standardized moment filters after temporal normalization filtering. Additional post-filter normalization applied. **a)** Low-contrast intrinsic optical signal images. **b)** Enhanced SD wavefront.

For near-infrared illumination, two approaches were used depending on the type of coherent illumination employed. For IOSI (low-coherence), the low vessel-contrast required only one additional artifact removal step. After the calculation of wavefronts, the baseline wavefront maps were subtracted to remove specular reflections (see Fig. 12). Conversely, for LSCI (high coherence), the high contrast of vessels requires the removal of a baseline edge map. Moreover, as the leading edge of the wavefront is less sharp for this modality, filtering must be applied without prior temporal averaging (data not shown).

## Discussion

Acute suppression of SD was observed with administration of low dosage of a nonsteroidal anti-inflammatory drug and to a lesser extent with an anti-malarial drug, both of which affect astroglial gap junctions. We demonstrated in Section 3.1 that MFA reduced SD-associated [K^+^]_e_-wave propagation and in Section 3.2 that MQH intermittently reduced propagation. Moreover, MQH led to a lag in the distal SD [K^+^]_e_ -waveform relative to its manifestation near the focal onset. We further demonstrated in Section 3.3 that the cerebral blood flow response to SD was significantly reduced after MFA but not MQH administration. Remarkably, these drugs were both able to induce the aforementioned acute effects at a concentration of 1 ​mg/kg. This is well below the recommended effective anti-malarial single dose of MQH at 10–30 ​mg/kg [44] and just below the maintenance levels of MFA used in clinical anti-cancer trials [45].

A single SD event can be tracked with reasonably high precision without invoking computational algorithms [30]. However, in practice the mapping of event wavefronts is limited by *1)* location dependent variability in parenchyma, *2)* variability in peak depolarization, and *3)* poor measurement dynamic range associated with the low-luminosity imaging conditions required for long duration data collection. In this work, we found both from temporal traces of potassium and spatial maps of AF changes that third order variation allowed for both consistent event onset alignment and noise robust tracking of the leading edge of SD wavefronts. The tracking of wavefront leading edges permitted more comprehensive analysis such as the mapping of focal velocity changes shown here in Section 3.6.

The suppression of SD has been demonstrated with other protocols and drugs. Vagus nerve stimulation has been shown to reduce SD susceptibility and propagation velocity [46]. There are a number of pharmacological agents which have been shown to have similar suppression-like effects. A summary of several novel therapeutic targets against SD was provided by Chen et al. 2017 [47]. Of the available options, few have limited toxicity side effects. However, characterizing the effects of concurrent application of these and other potential therapeutics may lead us to better drug combinations. Here, we have demonstrated that the suppression of SD ​amplitude can occur independently from reductions in SD velocity. Our comprehensive imaging provides a foundation for expanded investigations of subtle region-specific effects.

The suppression of SD may be either because the threshold for induction has changed (through enhanced potassium buffering) or the neural tissue is unable to regenerate enough intracellular ATP to sustain SD associated neural spiking [48]. The former being the ideal we seek in a clinical setting and the latter exacerbating the damage we sought to prevent with event suppression. We suspect from the absence of changes in baseline blood flow and oxygenation that tissue viability was preserved. However, increases in the AF background suggest a high concentration of the oxidized lower energy state of FAD.

It has been previously shown that mild acidosis delays hypoxia-induced spreading depression [49] and also can block gap junctional communication [50,51]. Severe acidosis is typically damaging. However, mild is potentially neuro-protective and, in the aforementioned study [49], neural recovery after spreading depression was improved by mild acidosis. Consequently, the delay in the propagating events we observed after MQH administration could be due to changes in tissue acidosis. Furthermore, others have observed the modulation of SD by changes in extracellular pH [52]. Specifically, acidosis was observed to decrease the velocity of SD which we also observed with MQH administration. However, they also observed a decrease in event duration whereas we did not. The experiments we performed could be conducted under conditions of hypoxia or with H^+^ sensitive electrodes to determine if this may be a component of the suppression observed. However, the most parsimonious explanation of the effects of these two drugs on SD propagation is their common blocking actions on astroglial gap junctional communication. Our chosen drugs have opposing effects on potassium outflux as MFA opens K_V_7.2 channels and MQH blocks K_V_7.1 channels. Although the nature of SD suppression was different between the two drugs, observing SD suppression with both drugs despite their opposite effects on potassium outflux supports the larger effect contribution is due to the blocking of gap-junctions. Moreover, the central role of gap-junctional coupling is supported by our prior work where a gap-junction opener and unrelated gap-junction blockers were found to increase and decrease, respectively, the velocity of SD propagation [1]. Assessment of the degree to which modulating outward potassium currents contributed to our observed suppression will require further investigation. However, any associated change in potassium outflux should produce a change in depolarization-induced activity leading to an opposing change in potassium outflux.

After SD, pial arterioles constrict by a mechanism mediated by prostanoids [53]. As nonsteroidal anti-inflammatory drugs prevent the formation of prostaglandins there is reason to suspect that these dynamics could be affected by MFA (as a non-selective COX-1/2 inhibitor). Further analysis is required to determine if our results are consistent with ​predicted trends from such modulation. The vasoconstriction observed distally is consistent with suppression of prostaglandin induced vasodilation. However, this is restricted to the event and no changes in baseline blood flow were observed.

The recording of baseline SD events prior to drug administration was required to correct for between experiment variation. However, this restriction complicates clinical translation by precluding investigation of preventative administration. Moreover, significant upregulation of several neurotransmitter receptors is observed following cortical spreading depression [54]. Consequently, the interpretation of changes observed with sequential SD events may not be solely attributed to the effect of the intervention despite being distinct from each other and sham experiments.

In conclusion, we applied potassium-selective electrode recording and multi-modal imaging *in vivo* to investigate the modulation of SD by acute administration of drugs known to affect astrocytic gap junctional coupling. We found that meclofenamic acid, which blocks Cx30 and Cx43 channels, consistently suppressed the propagation of SD. The suppression of propagation was followed by a more gradual suppression of SD induction. The blood flow response was also reduced with regions distal to the SD induction showing greater consistency. The administration of MQH, which blocks Cx43 channels, increased the latency of potassium and blood flow changes distal to the site of induction. Furthermore, using a novel wavefront localization technique we found MQH administration reduced SD propagation velocity. Further studies are required to assess the longitudinal efficacy and safety of these systemically administrable drugs as a treatment for SD as a clinical co-morbidity.

## Declaration of competing interest

The authors declare the following financial interests/personal relationships which may be considered as potential competing interests: Mathieu Charveriat reports a relationship with Theranexus that includes board membership. This work was partially supported by Theranexus Company. The author DR was directly funded by Theranexus for one year.
