# Supplementary material for: Spreading depolarization suppression from inter-astrocytic gap junction blockade assessed with multimodal imaging and a novel wavefront detection scheme
Source: Neurotherapeutics. 2023 Dec 19;21(1):e00298. doi: 10.1016/j.neurot.2023.10.008 (PMC10903093; doi:10.1016/j.neurot.2023.10.008)
Supplement: Multimedia component 1 [file mmc1.docx]

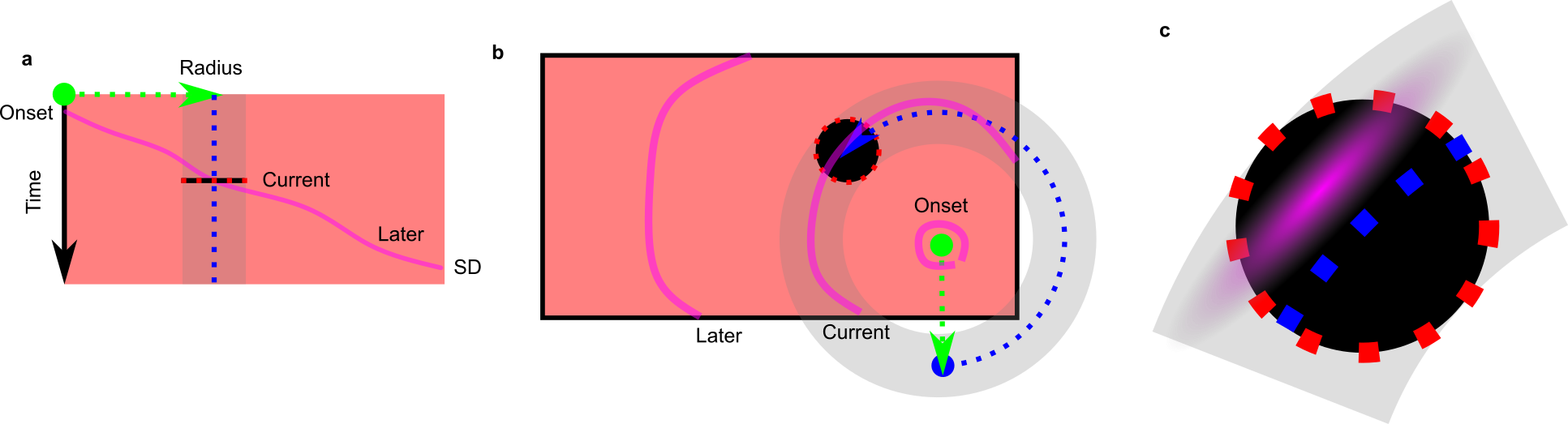


Figure **S**1: Visualization of azimuthal parametrization algorithm. **a)** Angular-projections over time of a propagating wavefront used for mean velocity estimation in this work. **b)** For a given time point in panel-a, an azimuthal circle corresponds to the radial segment about the wavefront. A bivariate moment filter can be rolled along the azimuthal-inscribed circle. **c)** For each point along the circle arbitrary moment-derived parameters can be computed: lead-lag (bivariate mean), orientation (covariance-matrix eigenvectors), curvature (derived from second and/or third order moments), symmetry (from skew), and visibility (from kurtosis-related parameters). Here we depicted a circle-leading wavefront with flat orientation, negligible curvature, with high symmetry and visibility.


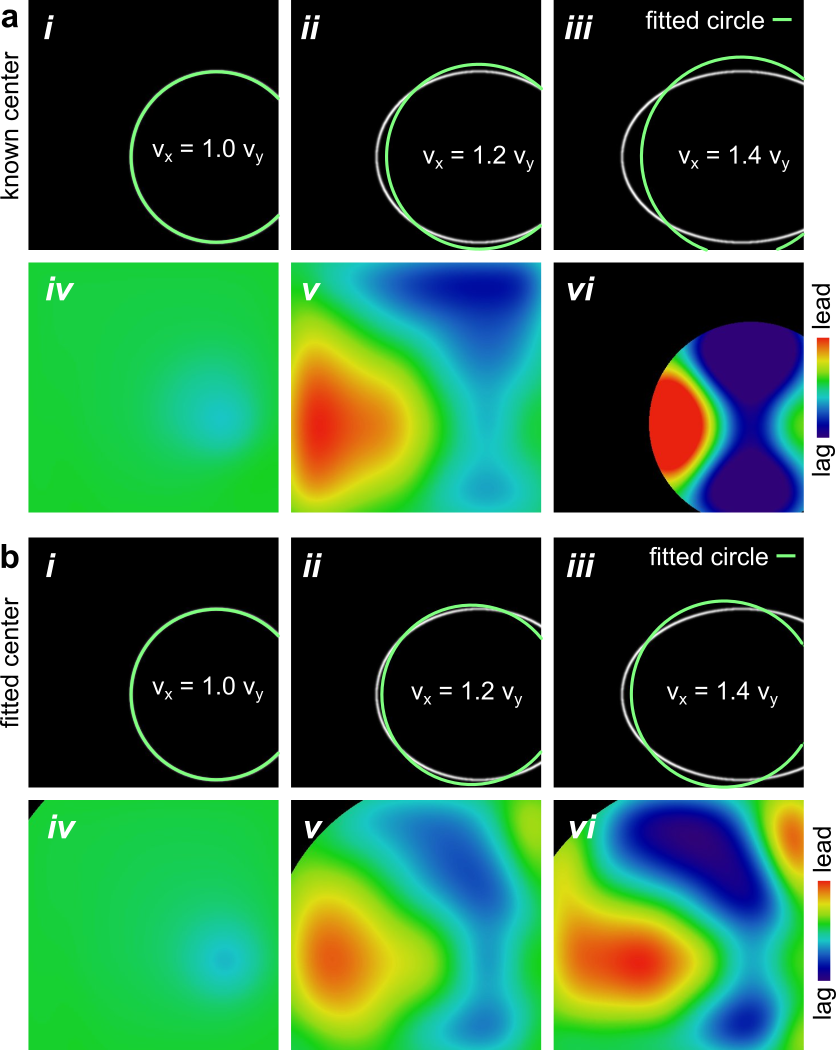


Figure **S**2: Wavefront deviation from circular propagation assessed with azimuthal parametrization scheme using simulated elliptical deviation. **a)** Radial least-squares fitting only, prior to azimuthal rolling lead-lag computation. **b)** Optimum least-squares circle fit, prior to azimuthal rolling lead-lag computation. In both panels a-b, *i-iii)* correspond to superposition upon increasingly elliptical simulated wavefronts. Running the azimuthal parametrization scheme at each time point enables the computation of wavefront lead-lag. These quantities can then be *iv-vi)* mapped back into their corresponding spatial coordinates.


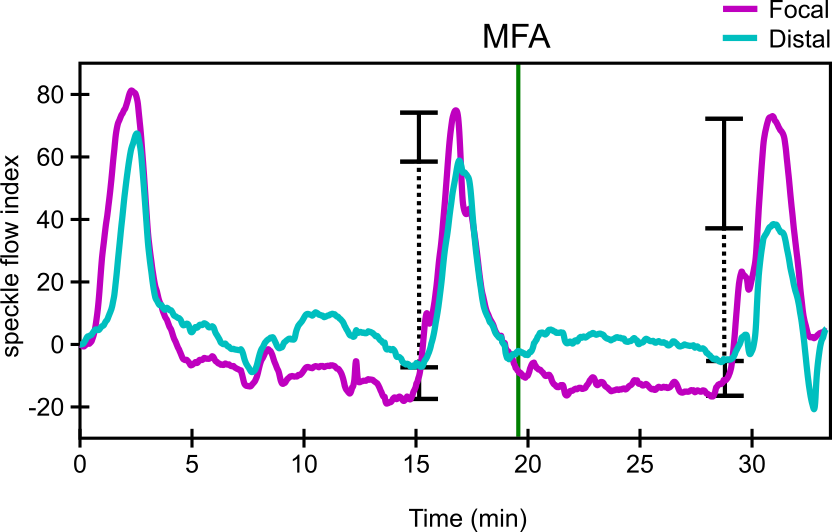


Figure **S**3: Focal and distal blood flow changes reflecting onset of distal SD suppression (vertical green line indicates MFA administration).


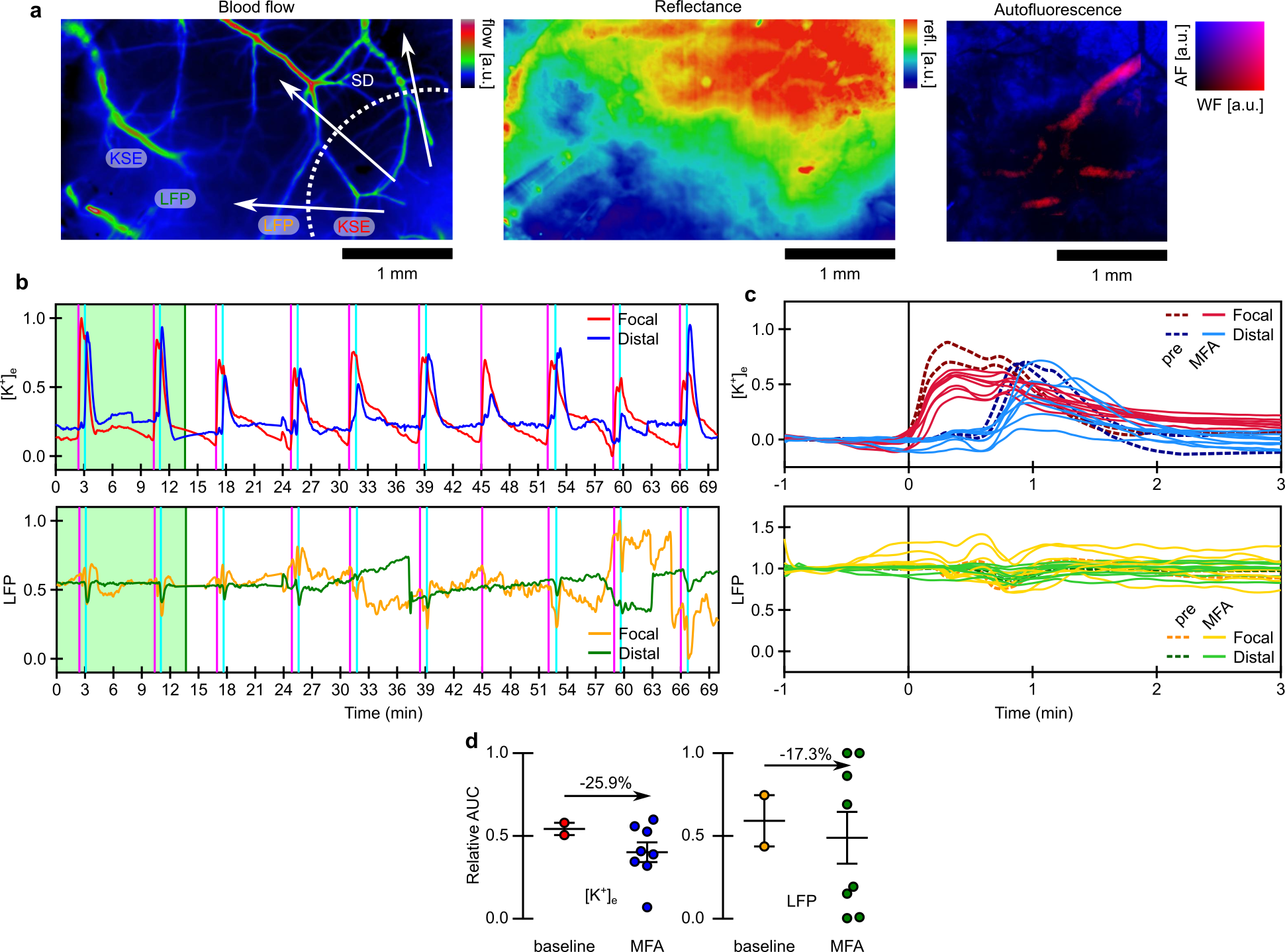


Figure **S**4: Suppression of SD with acute MFA administration (individual example).

**a)**Blood flow map from LSCI, with IOSI and AF maps acquired concurrently. The LFP and KSE locations are indicated (label colors correspond to panel-b traces). **b)** Recording of [K^+^]_e_ and LFP both adjacent and distal to the sight of SD induction (shaded green epoch is before MFA administration). Vertical lines indicate SD onset as classified by the individual [K^+^]_e_ recordings (adjacent/magenta and distal/cyan). **c)** Event onset overlaid traces synchronized to onset as defined by adjacent potassium trace (dashed traces represent pre-MFA administration). **d)** Treatment-induced change in the relative AUC between focal and distal [K^+^]_e_ and LFP recording. Error indicated by mean ± s.e.m.


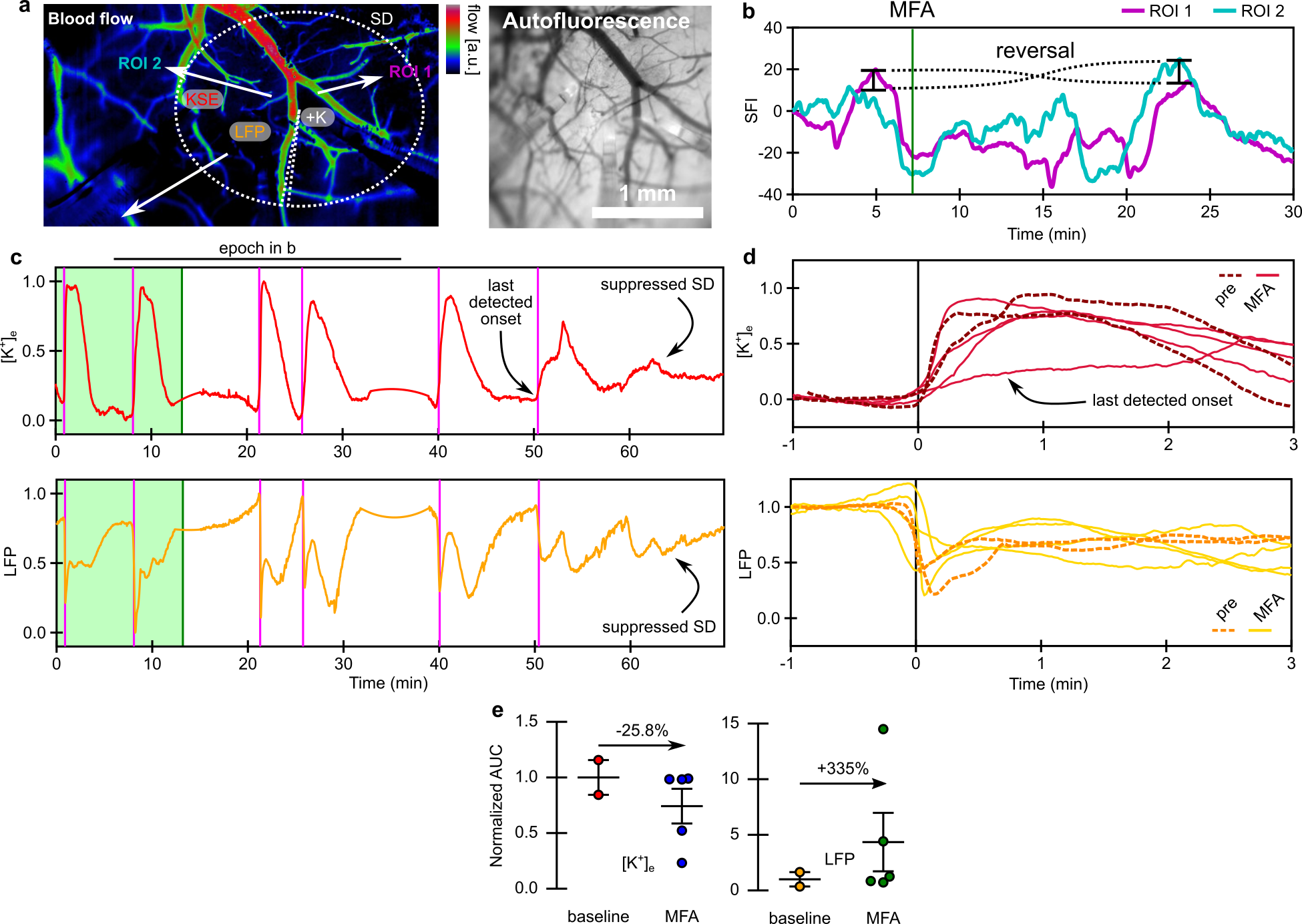


Figure **S**5: Example of the suppression of SD induction with acute MFA administration. **a)** Blood flow map from laser speckle flowmetry and AF map acquired concurrently. Electrode locations are indicated and reflect color correspondence of the traces below. **b)** Focal and distal blood flow changes reflecting suppressed depolarization event and a non-propagating depolarization event (vertical green line indicates MFA administration). **c)**Recording of [K^+^]_e_ and LFP both adjacent and distal to the sight of depolarization induction (shaded green epoch is before MFA administration). Vertical lines indicate the onset of events as classified by the individual potassium concentration recordings (adjacent/magenta and distal/cyan). **d)** Event onset overlaid traces synchronized to onset as defined by adjacent potassium trace (dashed traces are before MFA administration). **e)** Change in focal event AUC for both [K^+^]_e_ and LFP recordings. Error indicated by mean ± s.e.m.


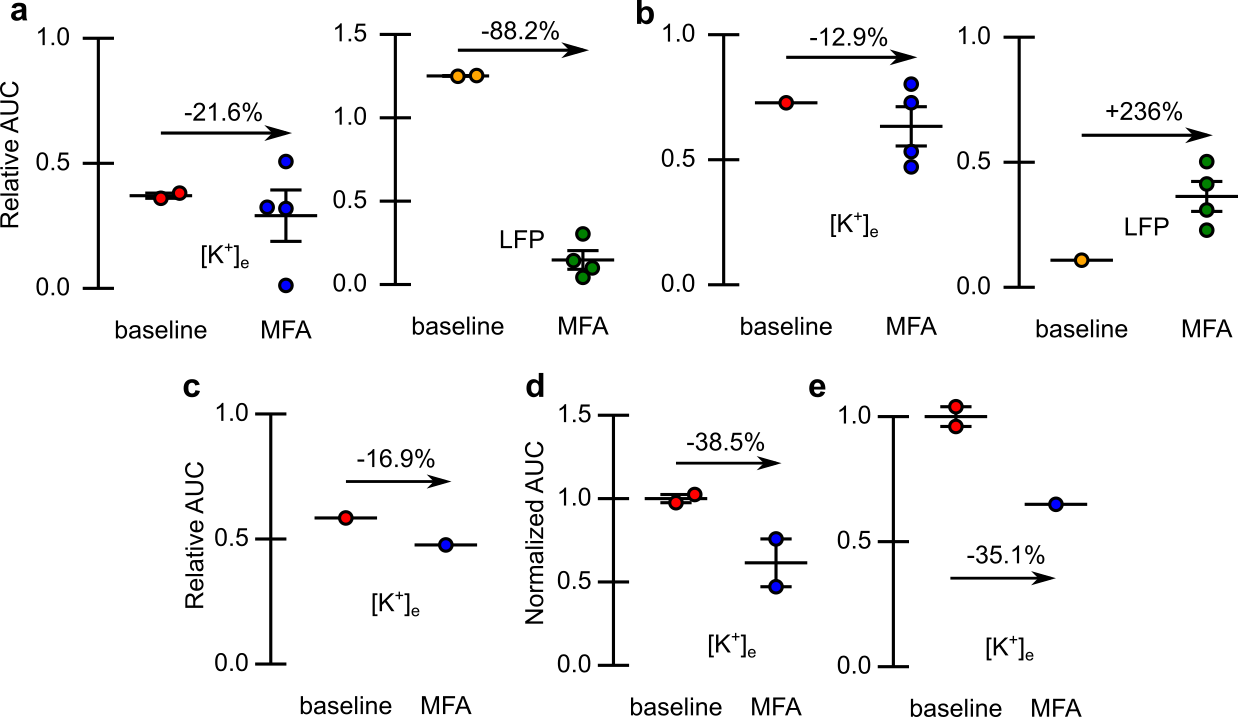


Figure **S**6: Additional examples of the suppression of SD with acute MFA administration. **a‑c)**Change in distal event AUC relative to focal for both [K^+^]_e_ and LFP recordings. **d-e)** Change in focal event AUC for both [K^+^]_e_ recordings. Error indicated by mean ± s.e.m.


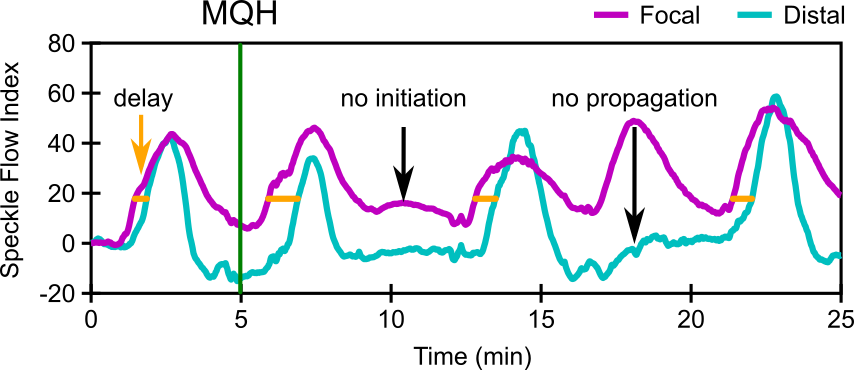


Figure **S**7: Focal and distal blood flow changes reflecting suppressed depolarization event and a non-propagating depolarization event. The horizontal bars indicate the delay between the blood flow response at onset distally (vertical green line indicates MQH administration).


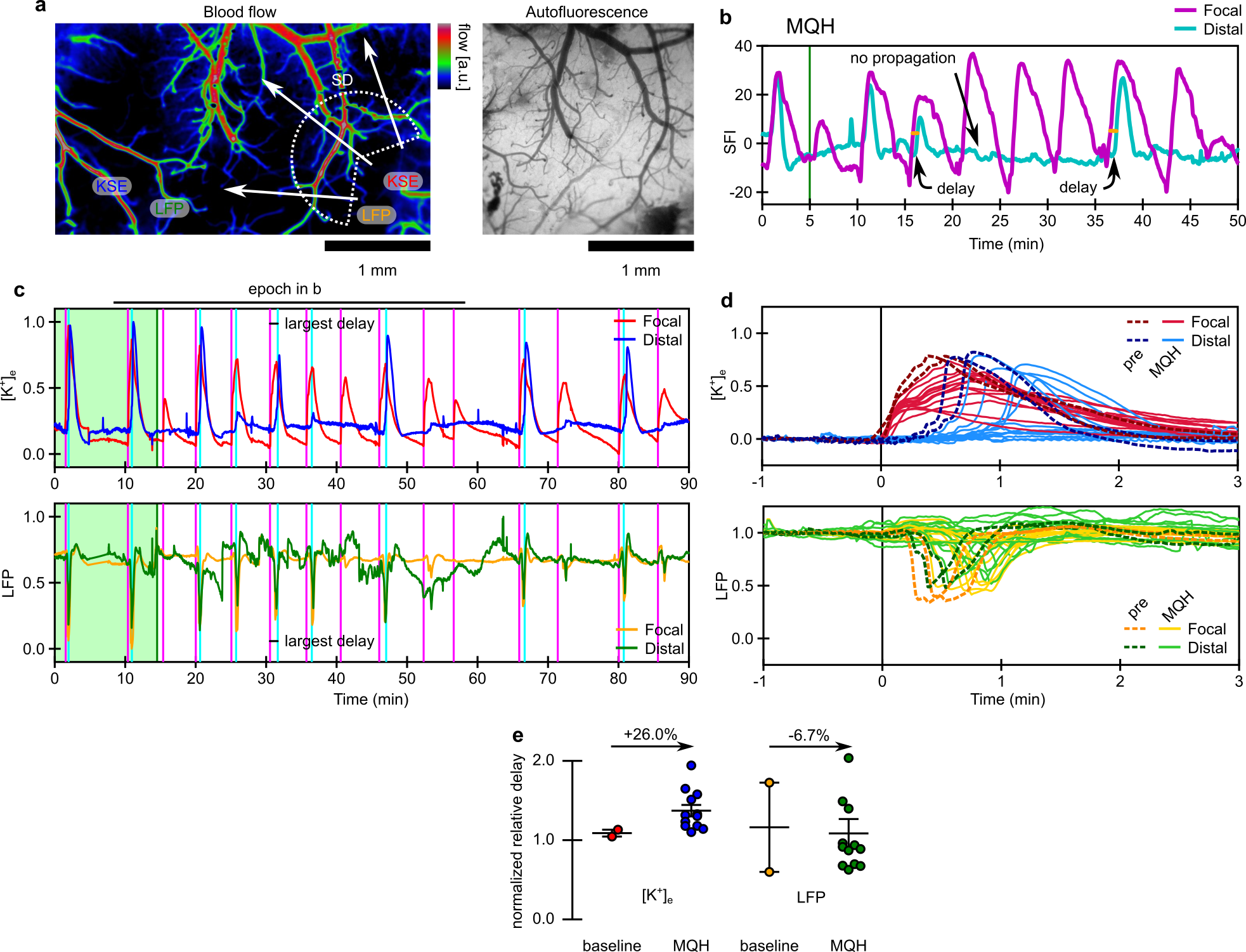


Figure **S**8: Modulation of SD propagation with acute MQH administration. **a)**Blood flow map from laser speckle flowmetry and AF map acquired concurrently. Electrode locations are indicated and reflect color correspondence of the traces below. **b)**Focal and distal blood flow changes reflecting suppressed depolarization event and a non-propagating depolarization event. The horizontal bars indicate the delay between the blood flow response at onset distally (vertical green line indicates MQH administration). **c)**Recording of [K^+^]_e_ and LFP both adjacent and distal to the sight of depolarization induction (shaded green epoch is before MQH administration). Vertical lines indicate the onset of events as classified by the individual [K^+^]_e_ recordings (adjacent/magenta and distal/cyan). **d)**Overlaid SD traces synchronized to onset as defined by induction-adjacent potassium trace (dashed traces are before MQH administration). The distal event traces after MQH administration clearly lag behind the focal event traces, as quantified by the curve-weighted expectation value. **e)**Quantification of treatment-induced change in the relative distal-to-focal event timing for both the [K^+^]_e_ and LFP recordings. Error indicated by mean ± s.e.m.


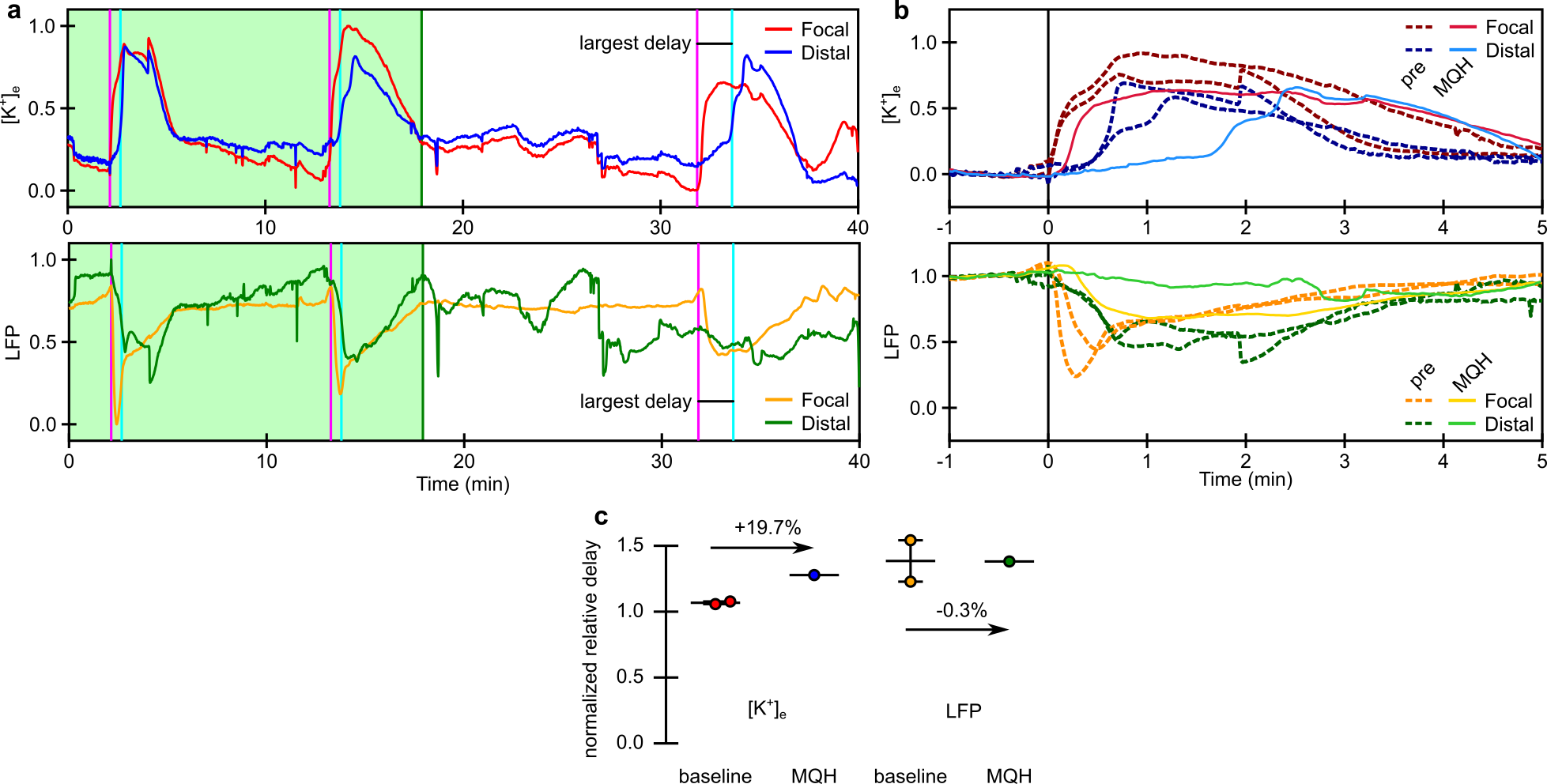


Figure **S**9: Modulation of SD propagation with acute MQH administration. **a)**Recording of [K^+^]_e_ and LFP both adjacent and distal to the sight of depolarization induction (shaded green epoch is before MQH administration). Vertical lines indicate the onset of events as classified by the individual [K^+^]_e_ recordings (adjacent/magenta and distal/cyan). **b)**Overlaid SD traces synchronized to onset as defined by induction adjacent potassium trace (dashed traces are before MQH administration). **c)**Quantification of treatment induced change in the relative distal-to-focal event timing for both the [K^+^]_e_ and LFP recordings. Error indicated by mean ± s.e.m.


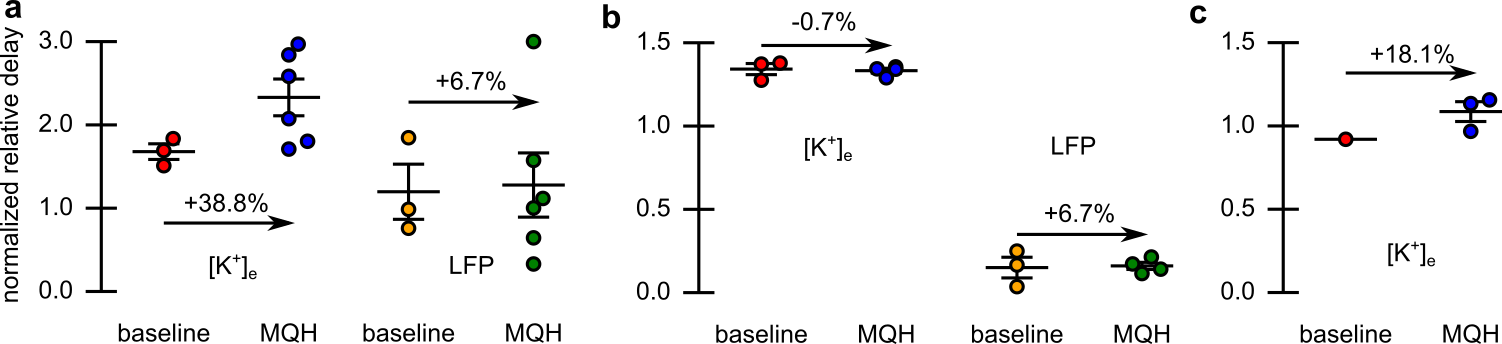


Figure **S**10: Additional examples of the modulation of SD propagation after acute MQH administration. **a‑c)**Change in distal event relative to focal for both KSE and LFP recordings. Error indicated by mean ± s.e.m.


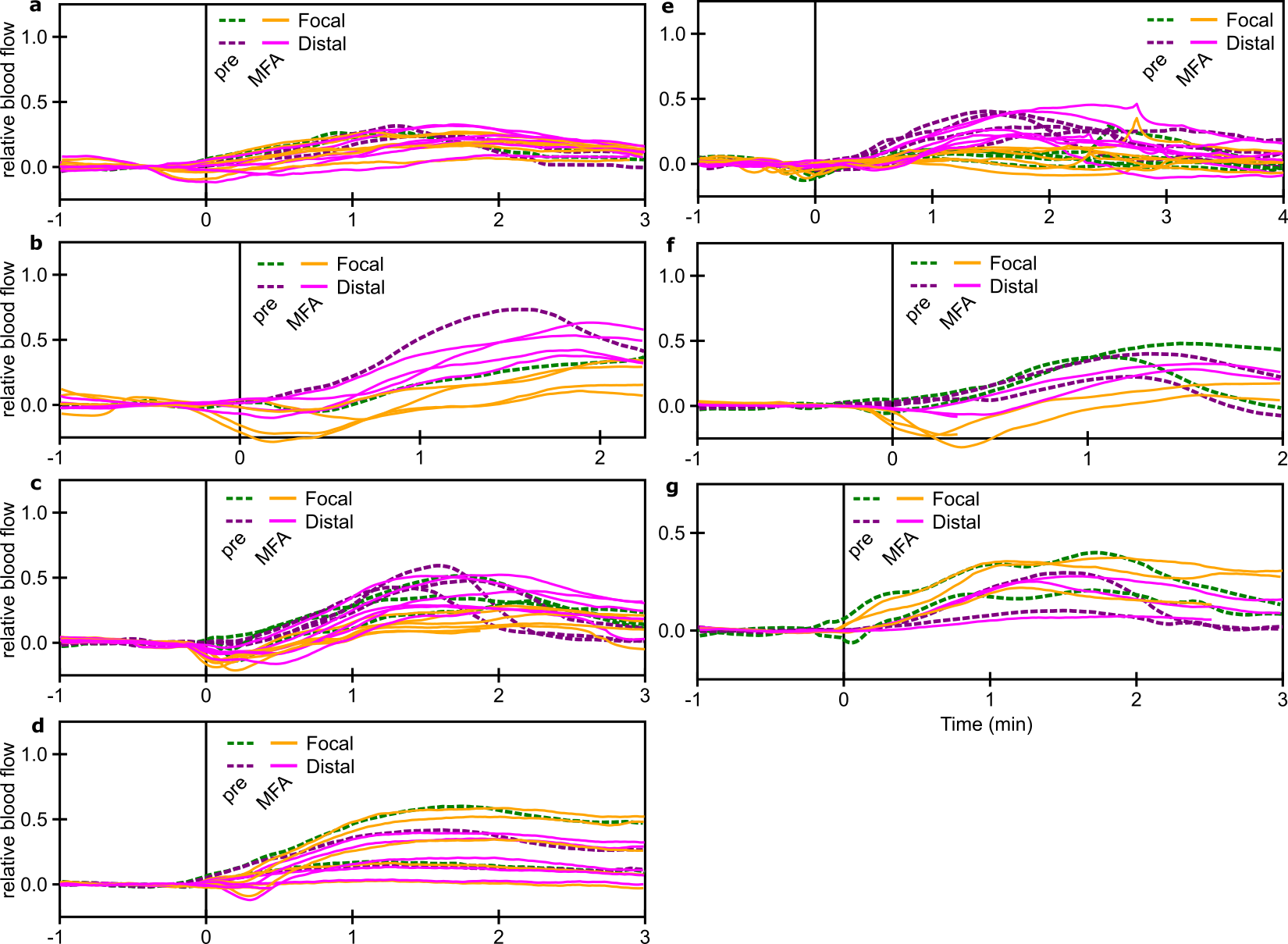


Figure **S**11: Relative blood flow synchronized to potassium SD-wave onset before and after MFA administration in both focal and distal regions of the cortex. **a-g)** Additional experiments used to derive statistics in Fig. 7b.


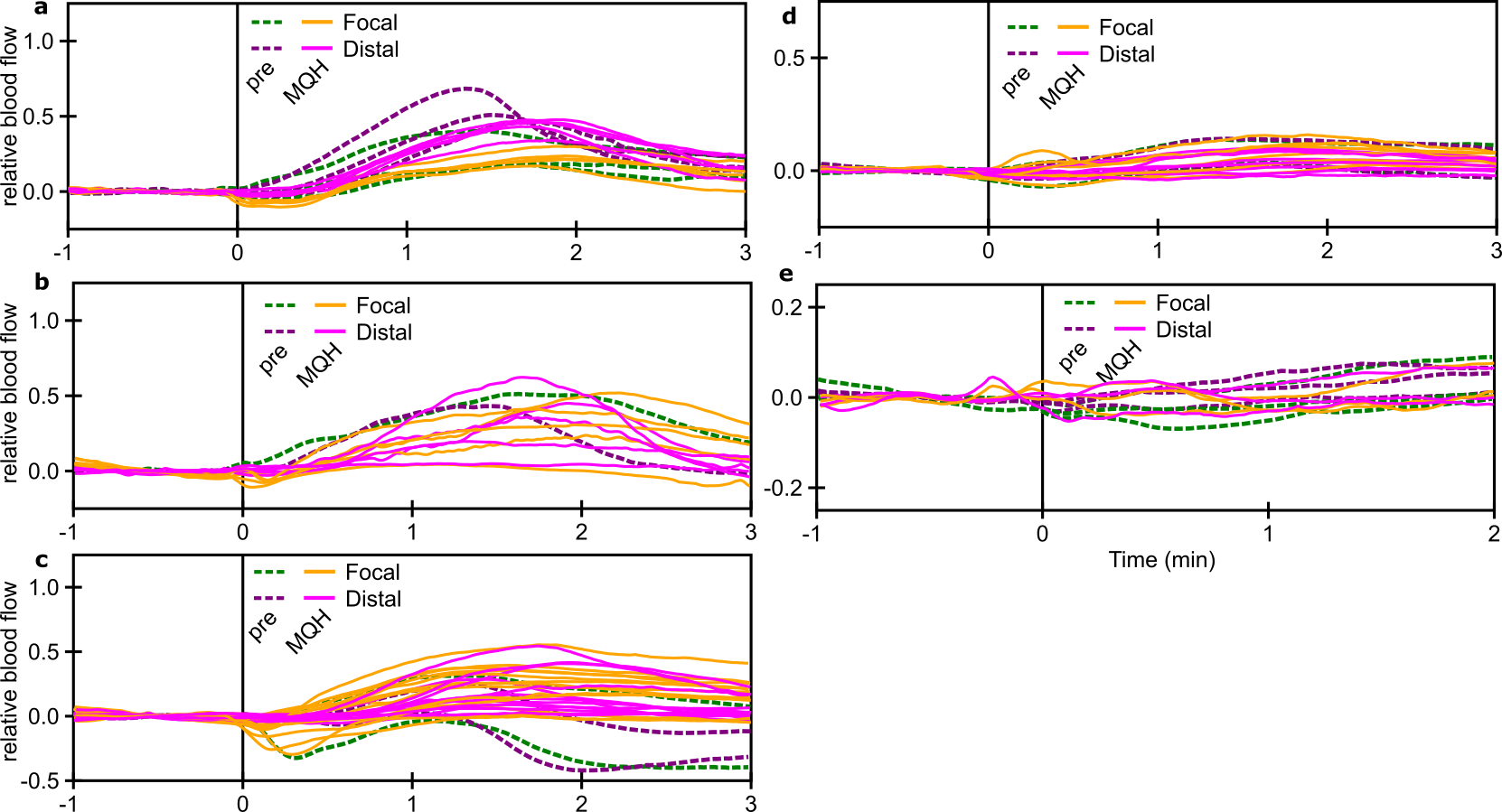


Figure **S**12: Relative blood flow synchronized to potassium SD-wave onset before and after MQH administration in both focal and distal regions of the cortex. **a-e)** Additional experiments used to derive statistics in Fig. 7d.
